# Disentangling polygenic associations between attention-deficit/hyperactivity disorder, educational attainment, literacy and language

**DOI:** 10.1038/s41398-018-0324-2

**Published:** 2019-01-24

**Authors:** Ellen Verhoef, Ditte Demontis, Stephen Burgess, Chin Yang Shapland, Philip S. Dale, Aysu Okbay, Benjamin M. Neale, Stephen V. Faraone, iPSYCH-Broad-PGC ADHD Consortium, Esben Agerbo, Thomas Damm Als, Marie Bækved-Hansen, Rich Belliveau, Anders D. Børglum, Jonas Bybjerg-Grauholm, Felecia Cerrato, Kimberly Chambert, Claire Churchhouse, Søren Dalsgaard, Mark J. Daly, Ditte Demontis, Ashley Dumont, Jacqueline Goldstein, Jakob Grove, Christine S. Hansen, Mads Engel Hauberg, Mads V. Hollegaard, David M. Hougaard, Daniel P. Howrigan, Hailiang Huang, Julian Maller, Alicia R. Martin, Joanna Martin, Manuel Mattheisen, Jennifer Moran, Ole Mors, Preben Bo Mortensen, Benjamin M. Neale, Merete Nordentoft, Jonatan Pallesen, Duncan S. Palmer, Carsten Bøcker Pedersen, Marianne Giørtz Pedersen, Timothy Poterba, Jesper Buchhave Poulsen, Stephan Ripke, Elise B. Robinson, F. Kyle Satterstrom, Christine Stevens, Patrick Turley, Raymond K. Walters, Thomas Werge, Evie Stergiakouli, George Davey Smith, Simon E. Fisher, Anders D. Børglum, Beate St Pourcain

**Affiliations:** 1 Language and Genetics Department, Max Planck Institute for Psycholinguistics, Nijmegen, The Netherlands; 2 International Max Planck Research School for Language Sciences, Nijmegen, The Netherlands; 3 The Lundbeck Foundation Initiative for Integrative Psychiatric Research, iPSYCH, Aarhus, Denmark; 4 Centre for Integrative Sequencing, iSEQ, Aarhus University, Aarhus, Denmark; 5 Department of Biomedicine-Human Genetics, Aarhus University, Aarhus, Denmark; 6 MRC Biostatistics Unit, University of Cambridge, Cambridge, UK; 7 Cardiovascular Epidemiology Unit, Department of Public Health and Primary Care, University of Cambridge, Cambridge, UK; 8 Speech and Hearing Sciences, University of New Mexico, Albuquerque, USA; 9 Department of Complex Trait Genetics, Vrije Universiteit Amsterdam, Amsterdam, The Netherlands; 10 Erasmus University Rotterdam Institute for Behavior and Biology, Rotterdam, The Netherlands; 11 Analytic and Translational Genetics Unit, Department of Medicine, Massachusetts General Hospital and Harvard Medical School, Boston, MA USA; 12 Stanley Center for Psychiatric Research, Broad Institute of Harvard and MIT, Cambridge, MA USA; 13 Program in Medical and Population Genetics, Broad Institute of Harvard and MIT, Cambridge, MA USA; 14 Departments of Psychiatry and Neuroscience and Physiology, SUNY Upstate Medical University, New York, USA; 15 MRC Integrative Epidemiology Unit, University of Bristol, Bristol, UK; 16 School of Oral and Dental Sciences, University of Bristol, Bristol, UK; 17 School of Social and Community Medicine, University of Bristol, Bristol, UK; 18 Donders Institute for Brain, Cognition and Behaviour, Radboud University, Nijmegen, The Netherlands; 19 National Centre for Register-based Research, Aarhus University, Aarhus, Denmark; 20 Centre for Integrated Register-based Research, Aarhus University, Aarhus, Denmark; 21 Center for Neonatal Screening, Department for Congenital Disorders, Statens Serum Institut, Copenhagen, Denmark; 22 Bioinformatics Research Centre, Aarhus University, Aarhus, Denmark; 23 Institute of Biological Psychiatry, MHC Sct. Hans, Mental Health Services Copenhagen, Roskilde, Denmark; 24 Genomics plc, Oxford, United Kingdom; 25 Department of Medical Epidemiology and Biostatistics, Karolinska Institutet, Stockholm, Sweden; 26 MRC Centre for Neuropsychiatric Genetics and Genomics, School of Medicine, Cardiff University, Cardiff, United Kingdom; 27 Centre for Psychiatry Research, Department of Clinical Neuroscience, Karolinska Institutet, Stockholm, Sweden; 28 Stockholm Health Care Services, Stockholm County Council, Stockholm, Sweden; 29 Psychosis Research Unit, Aarhus University Hospital, Risskov, Denmark; 30 Mental Health Services in the Capital Region of Denmark, Mental Health Center Copenhagen, University of Copenhagen, Copenhagen, Denmark; 31 Department of Psychiatry, Charite Universitatsmedizin Berlin Campus Benjamin Franklin, Berlin, Germany; 32 Department of Epidemiology, Harvard Chan School of Public Health, Boston, MA USA; 33 Department of Clinical Medicine, University of Copenhagen, Copenhagen, Denmark

## Abstract

Interpreting polygenic overlap between ADHD and both literacy-related and language-related impairments is challenging as genetic associations might be influenced by indirectly shared genetic factors. Here, we investigate genetic overlap between polygenic ADHD risk and multiple literacy-related and/or language-related abilities (LRAs), as assessed in UK children (*N* ≤ 5919), accounting for genetically predictable educational attainment (EA). Genome-wide summary statistics on clinical ADHD and years of schooling were obtained from large consortia (*N* ≤ 326,041). Our findings show that ADHD-polygenic scores (ADHD-PGS) were inversely associated with LRAs in ALSPAC, most consistently with reading-related abilities, and explained ≤1.6% phenotypic variation. These polygenic links were then dissected into both ADHD effects shared with and independent of EA, using multivariable regressions (MVR). Conditional on EA, polygenic ADHD risk remained associated with multiple reading and/or spelling abilities, phonemic awareness and verbal intelligence, but not listening comprehension and non-word repetition. Using conservative ADHD-instruments (*P*-threshold < 5 × 10^−8^), this corresponded, for example, to a 0.35 SD decrease in pooled reading performance per log-odds in ADHD-liability (*P* = 9.2 × 10^−5^). Using subthreshold ADHD-instruments (*P*-threshold < 0.0015), these effects became smaller, with a 0.03 SD decrease per log-odds in ADHD risk (*P* = 1.4 × 10^−6^), although the predictive accuracy increased. However, polygenic ADHD-effects shared with EA were of equal strength and at least equal magnitude compared to those independent of EA, for all LRAs studied, and detectable using subthreshold instruments. Thus, ADHD-related polygenic links with LRAs are to a large extent due to shared genetic effects with EA, although there is evidence for an ADHD-specific association profile, independent of EA, that primarily involves literacy-related impairments.

## Introduction

Children with Attention-Deficit/Hyperactivity Disorder (ADHD) often experience difficulties mastering literacy-related and/or language-related abilities (LRAs)^1–3^. It has been estimated that up to 40% of children diagnosed with clinical ADHD also suffer from reading disability (RD, also known as developmental dyslexia) and vice versa^4^. The spectrum of affected LRAs in ADHD may, however, also include writing^5,6^, spelling^7,8^, syntactic^9,10^ and phonological^9,10^ abilities. Both clinical ADHD and RD are complex childhood-onset neurodevelopmental conditions that affect about 5% and 7% of the general population, respectively^11,12^. ADHD is characterised by hyperactive, inattentive and impulsive symptoms^13^, whereas decoding and/or reading comprehension deficits are prominent in individuals with RD^14^.

To interpret the comorbidity of ADHD and RD, a multiple-deficit model including shared underlying aetiologies has been proposed, involving both genetic and environmental influences^15^. This model is supported by twin studies suggesting that the co-occurrence of ADHD symptoms and reading deficits is, to a large extent, attributable to shared genetic influences^16–18^. Further twin research suggests that the genetic covariance between reading difficulties and ADHD is largely independent of genetic factors shared with IQ^19^, although it is not known whether these findings extend to a wider spectrum of LRAs, beyond reading abilities. Furthermore, the interpretation of polygenic ADHD-LRA overlap using markers on genotyping arrays is more challenging. There is strong evidence that genetically predicted educational attainment (EA)^20^ shares genetic variability with both ADHD^21^ and reading abilities^22,23^. Genetically predicted EA is a genetic proxy of cognitive abilities, but also socioeconomic status^20^ including, for example, associations with maternal smoking during pregnancy, parental smoking, household income or watching television^24^. Thus, observed genetic associations between ADHD and reading abilities may solely reflect shared genetic variation with EA, but not any other, more specific neuro-cognitive mechanisms. In other words, polygenic associations might be inflated or even induced^25^ by genetically predictable traits that are related to both, ADHD and reading abilities (or other LRAs).

Here, we (a) study polygenic links between clinical ADHD and a wide range of population-ascertained literacy-related and language-related measures as captured by common variation, (b) evaluate to what extent such links reflect a shared genetic basis with EA and (c) assess whether there is support for shared genetic factors between clinical ADHD and LRAs conditional on genetically predicted EA.

Studied ADHD polygenic scores (ADHD-PGS) are based on ADHD genome-wide association study (GWAS) summary statistics from two large independent ADHD samples, the Psychiatric Genomics Consortium (PGC) and the Danish Lundbeck Foundation Initiative for Integrative Psychiatric Research (iPSYCH), and a combination thereof. Associations between ADHD-PGS and a wide spectrum of population-based literacy-related and language-related measures related to reading, spelling, phonemic awareness, listening comprehension, non-word repetition and verbal intelligence skills, are examined in a sample of children from the UK Avon Longitudinal Study of Parents and Children (ALSPAC). Applying multivariable regression (MVR) techniques, analogous to Mendelian Randomisation (MR) approaches^26^, we report here disentangled associations between polygenic ADHD risk and LRA measures and estimate effects independent of and shared with genetically predicted years of schooling, using summary statistics from the Social Science Genetic Association Consortium (SSGAC).

## Methods and materials

### Literacy-related and language-related abilities in the general population

LRAs were assessed in children and adolescents from ALSPAC, a UK population-based longitudinal pregnancy-ascertained birth cohort (estimated birth date: 1991–1992, Supplementary Information)^27,28^. Ethical approval was obtained from the ALSPAC Law-and-Ethics Committee (IRB00003312) and the Local Research-Ethics Committees. Written informed consent was obtained from a parent or individual with parental responsibility and assent (and for older children consent) was obtained from the child participants.

#### Phenotype information

Thirteen measures capturing LRAs related to reading, spelling, phonemic awareness, listening comprehension, non-word repetition and verbal intelligence scores were assessed in 7 to 13 year-old ALSPAC participants (*N* ≤ 5919, Table 1) using both standardised and ALSPAC-specific instruments. Detailed descriptions of all LRA measures are available in Table 1 and the Supplementary Information.

**Table 1:** Literacy-related and language-related abilities in the Avon Longitudinal Study of Parents and Children

LRA (psychological instrument)	Mean Score (SE)	Mean Age (SE)	*N* (%males)	LRA combinations	
Reading accuracy and comprehension (WORD^69^), words	28.44 (9.24)	7.53 (0.31)	5891 (50.6)	---------------------Reading----------------	--------------------------- --------Global LRAs------------- -----------------------------------
Reading accuracy (ALSPAC specific: NBO^70^), words	7.55 (2.44)	9.87 (0.32)	5738 (49.3)		
Reading speed^a^ (NARA II^71^), passages	105.50 (12.47)	9.88 (0.32)	5189 (49.1)		
Reading accuracy^a^ (NARA II^71^), passages	104.11 (13.62)	9.88 (0.32)	5201 (49.1)		
Reading speed (TOWRE^72^), words	82.58 (10.28)	13.83 (0.20)	4247 (48.4)		
Non-word reading accuracy (ALSPAC specific: NBO^70^)	5.24 (2.48)	9.87 (0.32)	5731 (49.2)		
Non-word reading speed (TOWRE^72^)	50.82 (9.38)	13.83 (0.20)	4237 (48.3)		
Spelling accuracy (ALSPAC specific: NB)	7.89 (4.39)	7.53 (0.31)	5800 (50.2)	-Spelling-	
Spelling accuracy (ALSPAC specific: NB)	10.27 (3.43)	9.87 (0.32)	5728 (49.2)		
Phonemic awareness (AAT^73^)	20.23 (9.51)	7.53 (0.31)	5919 (50.6)		
Listening comprehension (WOLD^74^)	7.50 (1.96)	8.63 (0.30)	5473 (49.9)		
Non-word repetition (CNRep^75^)	7.26 (2.51)	8.63 (0.30)	5464 (49.9)		
Verbal intelligence^a^ (WISC-III^76^)	107.85 (16.74)	8.64 (0.31)	5456 (49.7)		

*Note*: Thirteen LRAs capturing aspects related to reading, spelling, phonemic awareness, listening comprehension, non-word repetition and verbal intelligence were assessed in 7 to 13 year-old ALSPAC participants using both standardised and ALSPAC-specific instruments (Supplementary Information)

*LRAs* literacy-related and language-related abilities, *WORD* Wechsler Objective Reading Dimension, *ALSPAC* Avon Longitudinal study of Parents and Children, *NBO* ALSPAC-specific assessment developed by Nunes, Bryant and Olson, *NARA II* The Neale Analysis of Reading Ability-Second Revised British Edition, *TOWRE* Test Of Word Reading Efficiency, *NB* ALSPAC-specific assessment developed by Nunes and Bryant, *AAT* Auditory Analysis Test, *WOLD* Wechsler Objective Language Dimensions, *CNRep* Children’s Test of Nonword Repetition, *WISC-III* Wechsler Intelligence Scale for Children III

^a^Scores were derived using age norms and adjusted for sex and principal components only before transformation

All LRA scores were rank-transformed to allow for comparisons of genetic effects across different psychological instruments with different distributions (Supplementary Information). Phenotypic correlations, using Pearson-correlation coefficients, were comparable for untransformed and rank-transformed scores (Table S1). To account for multiple testing, we estimated the effective number of phenotypes studied using Matrix Spectral Decomposition^29^(MatSpD), revealing seven independent measures (experiment-wide error rate of 0.007).

For sensitivity analysis, we excluded 188 children with an ADHD diagnosis at age 7, based on the Development and Wellbeing Assessment (DAWBA)^30^ (Supplementary Information).

#### Genetic analyses

ALSPAC participants were genotyped using the Illumina HumanHap550 quad chip genotyping platforms, and genotypes were called using the Illumina GenomeStudio software. Genotyping, imputation and genome-wide association analysis details are described in the Supplementary Information and Table 2.

**Table 2:** Sample description

Phenotype	Sample	Source	Ethnicity	Imputation reference panel	*N*
LRAs	ALSPAC	General population	White European	HRC r1.1	≤5891
ADHD	PGC	Clinical sample	Predominantly white European	HapMap phase 3	16,203 (*N*_cases_ = 4163)
ADHD	iPSYCH	Clinical sample	White European	1000 Genomes phase 3	37,076 (*N*_cases_ = 14,584)
ADHD	PGC + iPSYCH (EUR)	Clinical sample	White European	1000 Genomes phase 3	53,293 (*N*_cases_ = 19,099)
ADHD	PGC + iPSYCH	Clinical sample	Predominantly white European	1000 Genomes phase 3	55,374 (*N*_cases_ = 20,183)
EA	SSGAC	Predominantly general population	White European	1000 Genomes phase 3^a^	326,041

^a^Predominantly 1000 Genomes phase 3 (20)

Abbreviations: *LRAs* literacy-related and language-related abilities, *ADHD* Attention-Deficit/Hyperactivity Disorder, *EA* educational attainment, *ALSPAC* Avon Longitudinal study of Parents and Children, *PGC* Psychiatric Genomics Consortium, *iPSYCH* The Lundbeck Foundation Initiative for Integrative Psychiatric Research, *EUR* European ancestry, *SSGAC* Social Science Genetic Consortium, *HRC* The Haplotype Reference Consortium

*Note*: There is no overlap between LRA, ADHD and EA samples

### Clinical ADHD summary statistics

*Psychiatric Genomics Consortium (PGC)*. GWAS summary statistics were obtained from a mega-analysis of clinical ADHD^31^, conducted by the PGC (4163 cases and 12,040 controls/pseudo-controls) (Table 2, Supplementary Information, www.med.unc.edu/pgc/).

*The Lundbeck Foundation Initiative for Integrative Psychiatric Research (iPSYCH)*. An independent set of ADHD GWAS summary statistics were accessed through the Danish iPSYCH project^32^ (14,584 ADHD cases, 22,492 controls) (Table 2, Supplementary Information), using samples from the Danish Neonatal Screening Biobank hosted by Statens Serum Institute^21,33^.

*Combined PGC and iPSYCH ADHD sample (PGC* *+* *iPSYCH)*. To maximise power, we also analysed meta-GWAS summary statistics from an ADHD sample containing both PGC and iPSYCH participants^21^ (20,183 cases, 35,191 controls/pseudo-controls) (Table 2, www.med.unc.edu/pgc/) and its European-only subset (*PGC* *+* *iPSYCH(EUR)*, 19,099 cases, 34,194 controls/pseudo-controls) (Table 2, www.med.unc.edu/pgc/).

Detailed sample descriptions are available in Table 2 and the Supplementary Information.

### Educational attainment summary statistics

GWAS summary statistics for EA^20^ (discovery and replication sample combined, excluding ALSPAC and 23andMe samples, *N* = 326,041) were obtained from the SSGAC consortium. EA was assessed as years of schooling^20^. A detailed sample description is available in Table 2 and the Supplementary Information.

### Genome-wide complex trait analysis

SNP-h^2^ and genetic correlations (*r*_g_) between LRAs were estimated using Restricted Maximum Likelihood (REML) analyses^34,35^ as implemented in Genome-wide Complex Trait Analysis (GCTA) software^36^, including individuals with a genetic relationship < 0.05^34^. For this study, we selected only LRAs with evidence for SNP-h^2^ and sample size *N* > 4000 (Table S2).

### Linkage disequilibrium score regression and correlation

Linkage Disequilibrium Score (LDSC) regression^37^ was used to distinguish confounding biases from polygenic influences by examining the LDSC regression intercept. Unconstrained LD-score correlation^38^ analysis was applied to estimate *r*_g_ (Supplementary Information).

### Polygenic scoring analyses

ADHD-PGS^39,40^ were created in ALSPAC using the independent PGC and iPSYCH GWAS summary statistics, and, to maximise power, also for GWAS summary statistics from the combined PGC + iPSYCH sample (Supplementary Information). ADHD-PGS have been previously linked to ADHD symptoms in ALSPAC participants^41^. Rank-transformed LRAs were regressed on Z-standardised ADHD-PGS (aligned to measure risk-increasing alleles) using ordinary least square (OLS) regression (R:stats library, Rv3.2.0). The proportion of phenotypic variance explained is reported as OLS-regression-R^2^. Beta-coefficients (β) for ADHD-PGS quantify here the change in standard deviation (SD) units of LRA performance per one SD increase in ADHD-PGS.

### Multivariable regression analysis

To study the genetic association between ADHD and LRAs conditional on genetic influences shared with EA, we applied MVR. This technique is analogous to MR methodologies^26^ and controls for collider bias^42^ through the use of GWAS summary statistics. Technically, it involves the regression of regression estimates from independent samples on each other^26^ (Supplementary Information). Within this study we use MVR without inferring causality due to violations of classical MR assumptions^26^ (see below).

*Genetic variant selection:* To disentangle ADHD-LRA associations, we selected two sets of instruments from the most powerful ADHD GWAS summary statistics (PGC + iPSYCH). The first set contained genome-wide significant variants (*P* < 5 × 10^−8^, conservative). The second set included variants passing a more lenient *P*-value threshold (*P* < 0.0015, subthreshold) to increase power, consistent with current guidelines for the selection of genetic instruments in MR (F-statistic < 10)^43^. All sets included independent (PLINK^44^ clumping: LD-r^2^ < 0.25, ± 500 kb), well imputed (INFO^45^ > 0.8) and common (EAF > 0.01) variants. This resulted in 15 conservative and 2689 < *N*_SNPs_ ≤ 2692 subthreshold ADHD-instruments (Table S8).

*Estimation of ADHD effects:* We extracted regression estimates for selected ADHD-instruments (conservative and subthreshold) from ADHD (PGC + iPSYCH), EA (SSGAC) and 13 LRA (ALSPAC) GWAS summary statistics. Analysing each set of variants independently, regression estimates for individual LRA measures (β) were regressed on both ADHD (β as lnOR) and EA regression estimates (β) using an OLS regression framework (R:stats library, Rv3.2.0). Outcomes were (1) a MVR regression estimate quantifying the change in SD units of LRA performance per log odds increase in ADHD risk conditional on years of schooling (ADHD effect independent of EA), and (2) a MVR regression estimate quantifying the change in SD units of LRA performance per year of schooling as captured by ADHD instruments (ADHD effect shared with EA). Latter MVR regression estimates capture here shared genetic effects between ADHD, EA and LRAs, including (1) genetic confounding (i.e., genetically predictable EA influences both ADHD and LRAs), (2) mediation (i.e., genetically predictable ADHD influences LRA indirectly through EA) and (3) biological pleiotropy (i.e., ADHD risk variants affect ADHD and EA through independent biological pathways). As ADHD risk and EA are inversely genetically related with each other^21^, they were reported to quantify change per missing year of schooling. To compare the magnitude of both MVR estimates, we also conducted analyses using fully standardised EA, ADHD and LRA regression estimates (Supplementary Information).

Finally, MVR regression estimates were meta-analysed and contrasted across reading-related, spelling-related and all LRA measures (excluding the composite measure verbal intelligence) (Table 1) using random-effects meta-regression, accounting for phenotypic correlations between LRAs (R:metafor library^46^, Rv3.2.0; Supplementary Information).

#### Sensitivity analyses

As the directionality of the relationship between ADHD, EA and LRAs cannot be inferred in this study, we also examined the genetic association between EA and LRAs, conditional on ADHD, using MVR. Two sets of EA instruments (conservative and subthreshold, Table S8) were selected from EA (SSGAC) GWAS summary statistics, analogous to the selection of ADHD instruments, and MVR was conducted as described above. Note that we did not create LRA instrument sets, as GWAS summary statistics of LRAs were underpowered.

### Attrition analysis

We carried out an *attrition* analysis in ALSPAC studying the genetic association between LRA-missingness and polygenic ADHD risk, using both polygenic scoring analyses and MVR (Supplementary Information).

## Results

### Genetic architecture of literacy-related and language-related abilities and clinical ADHD

Phenotypic variation in literacy-related and language-related measures (Table 1), including reading abilities (comprehension, accuracy and speed) assessed in words/passages and non-words, spelling abilities (accuracy), phonemic awareness, listening comprehension, non-word repetition and verbal intelligence scores, can be tagged by common variants, with SNP-h^2^ estimates between 0.32 (SE = 0.07, non-word repetition age 8) and 0.54 (SE = 0.07, verbal intelligence age 8) (Table S2; GCTA-based and LDSC-based estimations). Importantly, all LRAs are phenotypically (Table S1) and genetically (Table S3) moderately to strongly interrelated. The observed LDSC-based evidence for genetic liability of clinical ADHD within the PGC (LDSC-h^2^ = 0.08(SE = 0.03)), iPSYCH (LDSC-h^2^ = 0.26(SE = 0.02)) and PGC + iPSYCH samples (Table S4) is consistent with previous reports^21^.

### Association between ADHD polygenic risk scores and literacy-realted and language-related abilities

**Fig. 1:**
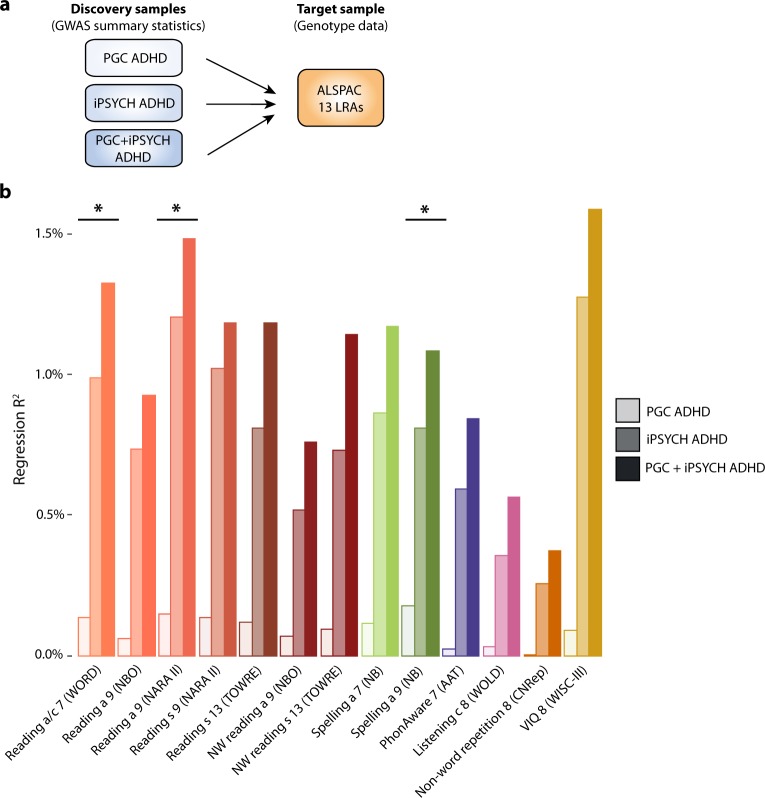
Phenotypic variance in literacy-related and language-related abilities explained by polygenic ADHD risk a accuracy, c comprehension, s speed, WORD Wechsler Objective Reading Dimension, NBO Nunes, Bryant and Olson (ALSPAC specific instrument), NARA II The Neale Analysis of Reading Ability-Second Revised British Edition, TOWRE Test Of Word Reading Efficiency, NW non-word, NB Nunes and Bryant (ALSPAC specific instrument), PhonAware phonemic awareness, AAT Auditory Analysis Test, WOLD Wechsler Objective Language Dimensions, CNRep Children’s Test of Nonword Repetition, VIQ verbal intelligence quotient, WISC-III Wechsler Intelligence Scale for Children III, PGC Psychiatric Genomics Consortium, iPSYCH The Lundbeck Foundation Initiative for Integrative Psychiatric Research, ADHD Attention-Deficit/Hyperactivity Disorder **a** Schematic representation of polygenic scoring analyses. ADHD polygenic scores were created in ALSPAC using PGC, iPSYCH and PGC + iPSYCH GWAS summary statistics. Rank-transformed LRAs were regressed on Z-standardised ADHD-PGS using ordinary least square regression. **b** Phenotypic variance in literacy-related and language-related abilities explained by polygenic ADHD risk. *Evidence for association between LRAs and polygenic ADHD risk as observed in PGC ADHD, iPSYCH ADHD and PGC + iPSYCH ADHD samples. Note that all LRAs were associated with polygenic ADHD risk in iPSYCH ADHD and PGC + iPSYCH ADHD passing the experiment-wide error rate (*P* < 0.007)

We observed robust evidence for an inverse genetic association between ADHD-PGS and reading accuracy/comprehension age 7 (PGC: OLS-R² = 0.1%, *P* = 4.6 × 10^-3^; iPSYCH: OLS-R² = 1.0%, *P* < 1 × 10^−10^), reading accuracy age 9 (PGC: OLS-R² = 0.1%, *P* = 5.7 × 10^−3^; iPSYCH: OLS-R² = 1.2%, *P* < 1 × 10^−10^), and spelling accuracy age 9 (PGC: OLS-R² = 0.2%, *P* = 1.5 × 10^−3^; iPSYCH: OLS-R² = 0.8%, *P* < 1 × 10^−10^) using independent ADHD discovery samples (Fig. 1, Table S5). The strongest evidence for association was observed when ADHD discovery samples were combined (PGC + iPSYCH; Fig. 1), including those of European ancestry only (PGC + iPSYCH(EUR)), with genetic trait-disorder overlap present for all LRAs studied (Table S5). For example, ADHD-PGS explain 1.49% phenotypic variation in reading accuracy age 9, translating into a genetic covariance of −0.11(95%-CI: −0.14; −0.09) (Supplementary Information). Polygenic scoring results are presented for a *P*-value threshold of 0.1, but other thresholds provided similar results (data not shown). Results were not affected by the exclusion of children with an ADHD diagnosis in ALSPAC (Table S6).

### Shared genetic liability between ADHD and LRA with EA

**Fig. 2:**
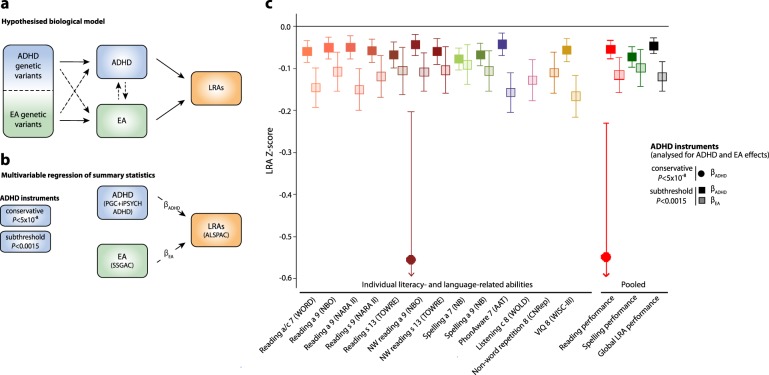
Genetic relationships between ADHD, educational attainment and literacy-related and language-related abilities ADHD Attention-Deficit/Hyperactivity Disorder, EA educational attainment, LRAs literacy and language-related abilities, PGC Psychiatric Genomics Consortium, iPSYCH The Lundbeck Foundation Initiative for Integrative Psychiatric Research; SSGAC Science Genetic Association Consortium, ALSPAC Avon Longitudinal Study of Parents And Children, MVR multivariable regression. **a** Hypothesised biological model of genetic relationships between ADHD, EA, and LRAs reflecting complex, pleiotropic and reciprocal genetic links that prevent causal inferences. **b** Schematic MVR model assessing polygenic ADHD-LRA overlap independent of and shared with genetic effects for EA. **c** MVR estimates of ADHD-specific effects independent of EA and ADHD effects shared with EA on LRAs using standardised ADHD instruments: Sets of conservative (*P* < 5 × 10^-8^) and subthreshold (*P* < 0.0015) ADHD instruments were extracted from ADHD (PGC + iPSYCH), EA (SSGAC) and LRAs (ALSPAC) GWAS summary statistics. ADHD-specific effects independent of EA (β_ADHD_) and ADHD effects shared with EA (β_EA_) on LRAs were estimated with MVRs. To compare the magnitude of β_ADHD_ and β_EA_, MVR analyses were conducted using standardised regression estimates (Supplementary Methods). β_ADHD_ estimates measure the change in LRA *Z*-score per *Z*-score in ADHD liability. β_EA_ estimates measure the change in LRA *Z*-scores per *Z*-score in missing school years. MVR estimates based on raw genetic effect estimates are provided in Table 3. Pooled estimates for reading, spelling and global LRA measures (Table 1) were obtained through random-effects meta-regression. Only effects passing the experiment-wide significance threshold (*P* < 0.007) are shown with corresponding 95% confidence intervals. There is no causality inferred

There was strong evidence for a moderate negative genetic correlation (*r*_g_ = –0.53(SE = 0.03), *P* < 1 × 10^−10^) between genetically predicted ADHD, as captured by the largest ADHD discovery sample (PGC + iPSYCH), and EA (LDSC-h^2^ = 0.11(SE = 0.004)), consistent with previous findings^21^. Likewise, LRAs were moderately to highly positively correlated with EA (e.g., reading speed age 13 *r*_g_ = 0.80(SE = 0.22), *P* = 3.0 × 10^−4^; Table S7), as previously reported^22,23^. Additionally, two independent variants reached genome-wide significance for both ADHD^21^ and EA^20^, consistent with biological pleiotropy (i.e., single genetic loci influencing multiple traits)^47^. These findings indicate complex, potentially reciprocal cross-trait relationships (Fig. 2a) and violate MR causal modelling assumptions^26^. Consequently, ADHD instruments are not valid MR instruments as they are not independent of EA.

### Multivariable regression analyses

To disentangle the genetic overlap of polygenic ADHD risk with literacy-related and language-related measures into ADHD genetic effects independent of and shared with EA, we applied MVR^26^ using ADHD instruments based on the most powerful ADHD discovery sample (PGC + iPSYCH) (Fig. 2b).

Using conservative ADHD instruments (Table S8), non-word reading accuracy at age 9 and pooled reading-related abilities were associated with polygenic ADHD risk, conditional on EA (Table 3). The latter translates into, for example, a decrease of 0.35 SD in pooled reading performance per log-odds increase in ADHD risk (β_ADHD_ = -0.35(SE = 0.09), *P* = 9.2 × 10^-5^, *P*_het_ = 0.19), an effect that was considerably stronger than for other LRAs (*P*_mod_ = 0.011, Table S10).

**Table 3:** Multivariable regression analysis of polygenic associations between ADHD and literacy-related and language-related abilities (raw estimates)

LRAs	ADHD-specific effects independent of EA (β_ADHD_)						EA genetic effects of ADHD-associated variants^a^ (β_EA_)					
	Conservative instruments (*P*_thr_ < 5 × 10^-8^)			Subthreshold instruments (*P*_thr_ < 0.0015)			Conservative instruments (*P*_thr_ < 5 × 10^-8^)			Subthreshold instruments (*P*_thr_ < 0.0015)		
	β (SE)	*P*	*P*_*het*_	β (SE)	*P*	*P*_*het*_	β (SE)	*P*	*P*_*het*_	β (SE)	*P*	*P*_*het*_
Reading a/c 7 (WORD)	–0.18 (0.13)	0.20	—	–0.03 (0.01)	1.4 × 10^−5^	—	−0.09 (1.19)	0.94	—	−0.63(0.10)	1.2 × 10^−9^	—
Reading a 9 (NBO)	−0.28 (0.09)	0.01	—	−0.03 (0.01)	2.1 × 10^−4^	—	1.33 (0.84)	0.14	—	−0.46 (0.10)	6.3 × 10^−6^	—
Reading s 9 (NARA II)	−0.29 (0.13)	0.04	—	−0.03 (0.01)	3.0 × 10^−5^	—	1.55 (1.16)	0.21	—	−0.51 (0.11)	2.5 × 10^−6^	—
Reading a 9 (NARA II)	−0.34 (0.12)	0.01	—	−0.03 (0.01)	3.8 × 10^−4^	—	1.57 (1.11)	0.18	—	−0.64 (0.11)	4.1 × 10^−9^	—
Reading s 13 (TOWRE)	−0.36 (0.15)	0.03	—	−0.04 (0.01)	2.7 × 10^−5^	—	2.06 (1.35)	0.15	—	−0.46 (0.12)	1.8 × 10^−4^	—
NW reading a 9 (NBO)	−0.37 (0.09)	0.002	—	−0.03 (0.01)	2.4 × 10^−4^	—	1.54 (0.85)	0.09	—	−0.46 (0.10)	6.1 × 10^−6^	—
NW reading s 13 (TOWRE)	−0.21 (0.17)	0.25	—	−0.03 (0.01)	2.4 × 10^−4^	—	1.43 (1.58)	0.38	—	−0.45 (0.12)	1.9 × 10^-4^	—
Spelling a 7 (NB)	−0.05 (0.12)	0.72	—	−0.05 (0.01)	1.4 × 10^−5^	—	-1.12 (1.13)	0.34	—	−0.63 (0.10)	1.2 × 10^−9^	—
Spelling a 9 (NB)	−0.23 (0.08)	0.01	—	−0.04 (0.01)	8.7 × 10^−7^	—	1.43 (0.72)	0.38	—	−0.45 (0.10)	1.3 × 10^−5^	—
PhonAware 7 (AAT)	−0.22 (0.15)	0.16	—	−0.02 (0.01)	0.002	—	−0.31 (1.33)	0.82	—	−0.67 (0.10)	< 1 × 10^−10^	—
Listening c 8 (WOLD)	−0.03 (0.08)	0.75	—	−0.02 (0.01)	0.02	—	−1.47 (0.70)	0.06	—	−0.55 (0.11)	4.5 × 10^−7^	—
Non-word repetition 8 (CNRep)	−0.14 (0.14)	0.34	—	−0.02 (0.01)	0.01	—	−0.16 (1.30)	0.90	—	−0.47 (0.11)	1.5 × 10^−5^	—
VIQ 8 (WISC-III)	−0.23 (0.15)	0.16	—	−0.03 (0.01)	5.0 × 10^−5^	—	−0.36 (1.37)	0.80	—	−0.71 (0.11)	< 1 × 10^−10^	—
Pooled reading	−0.35 (0.09)	9.2 × 10^−5^	0.19	−0.03 (0.01)	1.4 × 10^−6^	0.79	1.67 (0.78)	0.03	0.31	−0.50 (0.09)	4.9 × 10^−8^	0.09
Pooled spelling	−0.18 (0.11)	0.10	0.03	−0.04 (0.01)	1.1 × 10^−8^	0.28	0.40 (1.25)	0.75	0.001	−0.42 (0.10)	1.3 × 10^−5^	0.39
Pooled LRAs	−0.18 (0.07)	0.01	0.005	−0.03 (0.01)	1.9 × 10^−6^	0.05	0.29 (0.69)	0.67	0.001	−0.49 (0.08)	< 1 × 10^−10^	0.004

*Note*: Sets of conservative (*P* < 5 × 10^−8^) and subthreshold (*P* < 0.0015) ADHD instruments were extracted from ADHD (PGC + iPSYCH), EA (SSGAC) and LRAs (ALSPAC) GWAS summary statistics. ADHD-specific effects independent of EA (β_ADHD_) and ADHD effects shared with EA (β_EA_) on LRAs were estimated with MVRs (Fig. 2b). ADHD effects shared with EA were assessed through EA genetic effect estimates of ADHD-associated variants and presented with respect to missing school years. β_ADHD_ quantifies the change in LRA *Z*-score per log odds increase in ADHD liability. β_EA_ quantifies the change in LRA *Z*-score per missing year of schooling. Pooled estimates for reading, spelling and global LRAs (Table 1) were obtained through random-effects meta-regression. Evidence for effect heterogeneity (*P*_het_) was monitored through Cochran’s Q-test. To compare effect sizes of β_ADHD_ and β_EA_, MVR was carried out using standardised genetic effect estimates for which results are provided in Fig. 2c.

*LRAs* literacy-related and language-related abilities, *ADHD* Attention-Deficit/Hyperactivity Disorder, *EA* educational attainment, *P*_thr_*P*-value threshold, *P*_het_ heterogeneity *P*-value, *a* accuracy, *c* comprehension, *s* speed, *WORD* Wechsler Objective Reading Dimension, *NBO* Nunes, Bryant and Olson (ALSPAC specific instrument), *NARA II* The Neale Analysis of Reading Ability-Second Revised British Edition, *TOWRE* Test Of Word Reading Efficiency, *NW* non-word, *NB* Nunes and Bryant (ALSPAC specific instrument), *PhonAware* phonemic awareness, *AAT* Auditory Analysis Test, *WOLD* Wechsler Objective Language Dimensions, *CNRep* Children’s Test of Nonword Repetition, *VIQ* verbal intelligence quotient, *WISC-III* Wechsler Intelligence Scale for Children III, *MVR* Multivariable regression

^a^ADHD genetic effects shared with EA as assessed through EA genetic effect estimates of ADHD-associated variants

Using subthreshold ADHD instruments (Table S8), polygenic ADHD effects on LRA performance, conditional on EA, were detectable for all reading-related and spelling-related measures, phonemic awareness and verbal intelligence, but not other LRAs such as listening comprehension and non-word repetition (Table 3). Evidence was strongest for pooled reading and spelling abilities (Table 3, minimum *P* = 1.1 × 10^−8^). However, observable effects were smaller in magnitude compared to those captured by conservative ADHD instruments with, for example, a 0.03 SD decrease in pooled reading performance per log-odds increase in ADHD risk (β_ADHD_ = -0.03(SE = 0.01), *P* = 1.4 × 10^−6^, Table 3). Comparing ADHD-specific effects on both reading and spelling with ADHD-specific effects on all other LRAs provided evidence for effect differences (*P*_mod_ = 0.016), with stronger ADHD effects on literacy-related abilities, in particular spelling (Table S10).

Polygenic ADHD effects that are shared with EA were identified for all LRAs studied using subthreshold, but not conservative ADHD instruments (Table 3). This translates into, for example, a further 0.50 SD units decrease in pooled reading performance per missing school year (β_EA_ = −0.50(SE = 0.09), *P* = 4.9 × 10^−8^, Table 3). Thus, the observed association between polygenic ADHD risk and listening comprehension and non-word repetition is fully attributable to genetic effects shared with EA (Table 3). Contrary to ADHD-specific effects, ADHD effects shared with EA showed no evidence for effect differences between literacy-related versus other LRAs (*P* = 0.31). Conducting MVR with fully standardised estimates showed that ADHD effects shared with EA were as large as or even larger compared to ADHD-specific effects (Fig. 2c, Table S9).

Using an analogous approach, we disentangled the genetic overlap between polygenic EA and LRAs into genetic EA effects independent of and shared with ADHD, based on EA instruments (Fig. S1). There was strong evidence for EA effects shared with ADHD using subthreshold, but not conservative EA instruments (Table S11). The magnitude of ADHD genetic effects shared with EA, captured by ADHD genetic instruments, compared to the magnitude of EA genetic effects shared with ADHD, captured by EA instruments, was largely consistent with each other in fully standardised analyses (Tables S9 and S11).

There was little evidence supporting the inclusion of regression intercepts in MVR that would imply additional genetic effect variation in LRAs estimates, not yet captured by either ADHD or EA effect estimates, based on the selected instruments. Therefore, all MVRs were performed using constrained intercepts^26^.

### Attrition in ALSPAC

Analyses of sample drop-out in ALSPAC, exemplified by missing reading accuracy and comprehension scores at age 7 (WORD), revealed a positive genetic association between missingness and polygenic ADHD risk (min *P* = 1.4 × 10^−8^, Supplementary Information, Table S12, Table S13).

## Discussion

This study identified strong and replicated evidence for an inverse association between polygenic ADHD risk and multiple population-based LRAs using a polygenic scoring approach. However, these associations involve shared genetic variation with genetically predictable EA. Accurate modelling of polygenic links using MVR techniques, conditional on EA, revealed an ADHD-specific association profile that primarily involves literacy-related impairments. Once shared genetic effects with EA were accounted for, polygenic ADHD risk was most strongly inversely associated with reading and/or spelling abilities, in addition to phonemic awareness and verbal intelligence, but not listening comprehension and non-word repetition abilities. Importantly, genetic overlap between polygenic ADHD risk and all of the LRAs studied was inflated by genetic effects shared with EA.

Using independent ADHD discovery samples, these findings show that genetic overlap between ADHD and literacy-related impairments observed in twin and family studies^16–18^ can be extended to genetic associations, as captured by common variation in general population samples. The identified association profile suggests that not only reading-related abilities (including both word and nonword reading skills), but also phonological and spelling-related abilities share genetic aetiology with ADHD. These interrelated LRAs may, as hypothesised for RD, arise from a phonological impairment^48,49^, which affects decoding and reading skills^50^, but also spelling abilities^51^. However, reading abilities can, once developed, also shape phonological skills^52^.

In addition, this study suggests that genetic associations between polygenic ADHD and LRAs reflect, at least partially, shared genetic influences with genetically predictable EA and that, equally likely, genetic associations between polygenic EA and LRAs share genetic influences with ADHD. The magnitude of these shared effects, modelled with different MVR approaches, was comparable with each other. This is consistent with reciprocal genetic influences between EA and ADHD (Fig. 2a) and supports an intergenerational multiple-deficit model proposed for reading disability^15,53^. Children growing up in disadvantaged environments, genetically predictable through polygenic EA scores^54^, might be more vulnerable to psychiatric illness including ADHD^55^ that affects, in turn, their LRAs. In addition, adolescents with ADHD might be more likely to leave school at an earlier age, with lower LRA performance and EA, and pass on an increased genetic load to their own children^56^.

Here, we demonstrate that disentangling multivariate genetic interrelationships between ADHD, EA and LRAs using MVR can aid the interpretation of genetic overlap, while controlling for collider bias^42^. However, using MVR, the detection of these polygenic associations was strongly governed by the choice of genetic variants. Conservative ADHD instruments identified large ADHD-specific effects on reading as a domain and little evidence for genetic effects that are shared with EA, although they had limited power^57^. They comprised 15 independent SNPs only, including variation within *FOXP2*, a gene that has been implicated in childhood apraxia of speech and expressive and receptive language impairments (http://omim.org/entry/602081)^58^. On the other hand, subthreshold instruments, including thousands of variants, tagged ADHD-specific polygenic links with LRAs (conditional on EA) with smaller effects, but with higher predictive accuracy. However, these instruments also captured shared genetic effects with EA, affecting polygenic links between ADHD and all of the LRAs studied. These shared genetic influences were of equal strength and at least equal magnitude compared to ADHD-LRA associations independent of EA. Contrary, a previous twin study showed that the genetic covariance between ADHD and reading difficulties was largely independent of genetic effects shared with IQ^19^, suggesting that our findings may also reflect socio-economic influences. Thus, in order to improve reading and, more generally, literacy-related deficits in children with ADHD, there is potentially a need for further intervention programmes targeting EA-independent underlying neurocognitive deficits, beyond general training programmes aiming at schooling outcomes^59^.

In general, our findings are consistent with an omnigenic^60^ model of complex trait architectures, compatible with a general factor model of psychopathology^61^, including ADHD^62^. The omnigenic model construes that only the largest-effect variants will reflect ADHD specificity, and may thus tag the most trait-specific associations between ADHD and reading, independent of EA. The majority of variants, however, will capture pleiotropic (omnigenic) influences pointing to highly interconnected neural networks^60^ that give rise to genetic confounding. Consequently, the majority of subthreshold variants, captured by both ADHD and EA subthreshold variants, are likely to represent highly powerful cross-trait genetic predictors that may enhance and induce genetic overlap.

Finally, the methodological framework within this work has not only relevance for studies investigating polygenic links between ADHD and LRAs, but for many studies examining multivariate trait interrelationships that involve shared genetic effects with a genetically predictable confounder. Specifically, our findings suggest that lower variant selection thresholds can introduce genetic variance sharing that is unspecific and needs to be accounted for before identified associations can be interpreted in terms of underlying mechanisms, including shared genetic aetiologies. This is especially important as current guidelines for studying polygenic links with allelic scores recommend aggregating genetic variants across less stringent significance thresholds to maximise genetic association between discovery and target samples^63,64^.

This study has several limitations. Firstly, ALSPAC, as other cohort studies, suffers from attrition^65,66^. Sensitivity analyses showed that this is unlikely to bias our findings based on conservative instruments. However, links identified using subthreshold ADHD variants, might have been underestimated given that individuals with a genetic predisposition to ADHD (but also smoking initiation, higher body mass index, neuroticism, schizophrenia and depression) are more likely to drop out^66^. Secondly, the strength of the genetic overlap between polygenic ADHD risk and LRAs may vary according to ADHD symptom domain levels, implicating especially inattentiveness^67^, as well as the nature of the literacy-related or language-related ability involved (as we observed evidence for effect heterogeneity when combining all LRAs). It is conceivable that also other verbal abilities, not investigated in this study, such as grammar, expressive vocabulary or pragmatic skills, may genetically overlap with ADHD. Furthermore, we only studied the extent to which shared genetic variance with EA affects the genetic association between ADHD and LRAs. However, we found little evidence for the presence of additional unaccounted for genetic influences using these instruments, i.e., effects that are not yet captured by either genetically predicted ADHD or EA. Finally, the power of available LRA GWAS summary statistics is still too low to generate genetic instruments supporting reverse models. Larger and more detailed clinical and population-based samples, as well as extensive multivariate variance analyses of the spectrum of LRAs (that are currently computationally expensive^68^) will help to further characterise the overlap between ADHD and literacy-related and language-related cognitive processes.

## Conclusion

Polygenic associations of clinical ADHD and a range of LRAs are to a large extent attributable to genetic effects that are also shared with EA, especially when investigated with genetic variants typically selected for polygenic scoring approaches. Adjusting for these unspecific genetic effects reveals an ADHD-specific association profile that primarily involves literacy-related impairments.

## Supplementary information

Supplementary Information
