# Supplementary material for: Disentangling polygenic associations between attention-deficit/hyperactivity disorder, educational attainment, literacy and language
Source: Transl Psychiatry. 2019 Jan 24;9:35. doi: 10.1038/s41398-018-0324-2 (PMC6345874; doi:10.1038/s41398-018-0324-2)
Supplement: Supplementary file 1 — Supplementary Information [file 41398_2018_324_MOESM1_ESM.docx]

**Supplementary Information**

Disentangling polygenic associations between Attention-Deficit/Hyperactivity Disorder, educational attainment, literacy and language

[Supplementary methods & analyses 3](#_Toc527532816)

[ALSPAC description 3](#_Toc527532817)

[Literacy- and language-related measures in ALSPAC 5](#_Toc527532818)

[ALSPAC trait transformation 9](#_Toc527532819)

[ADHD cases within ALSPAC 10](#_Toc527532820)

[ALSPAC genome-wide analyses 11](#_Toc527532821)

[Clinical ADHD summary statistics 12](#_Toc527532822)

[Linkage Disequilibrium score regression and correlation 14](#_Toc527532823)

[Polygenic scoring analyses 15](#_Toc527532824)

[Overlap in genome-wide significant association signals between ADHD and educational attainment 16](#_Toc527532825)

[Multivariable regression 17](#_Toc527532826)

[Meta-regression across literacy- and language-related abilities 18](#_Toc527532827)

[Standardisation of genetic effect estimates 19](#_Toc527532828)

[Attrition analysis in ALSPAC 21](#_Toc527532829)

[Web resources 22](#_Toc527532830)

[Supplementary References 23](#_Toc527532831)

[Supplementary Tables 26](#_Toc527532832)

[Table S1: Phenotypic correlations among literacy- and language-related measures 26](#_Toc527532833)

[Table S2: SNP-heritability estimates for literacy- and language-related measures 28](#_Toc527532834)

[Table S3: Genetic correlations among literacy- and language-related measures 29](#_Toc527532835)

[Table S4: SNP-heritability estimates for clinical ADHD and educational attainment 30](#_Toc527532836)

[Table S5: Association of polygenic ADHD risk scores with literacy- and language-related measures 31](#_Toc527532837)

[Table S6: Association of polygenic ADHD risk scores with literacy- and language-related measures excluding ADHD children 33](#_Toc527532838)

[Table S7: Genetic correlations of literacy- and language-related measures with educational attainment 35](#_Toc527532839)

[Table S8: Selection of ADHD and EA instruments 36](#_Toc527532840)

[Table S9: Multivariable regression analysis of polygenic associations between ADHD and literacy- and language-related abilities (standardised) 37](#_Toc527532841)

[Table S10: Comparison of ADHD-specific MVR effects on literacy-related abilities versus other LRAs 39](#_Toc527532842)

[Table S11: Multivariable regression analysis of polygenic associations between EA and literacy- and language-related abilities (standardised) 40](#_Toc527532843)

[Table S12: Association between polygenic ADHD risk and sample-dropout 42](#_Toc527532844)

[Table S13: Multivariable regression analysis of polygenic associations between ADHD, sample drop-out and literacy- and language-related abilities 43](#_Toc527532845)

[Supplementary Figures 44](#_Toc527532846)

[Figure S1: Multivariable regression analysis of polygenic associations between EA and literacy- and language-related abilities 44](#_Toc527532848)

# Supplementary methods & analyses

## ALSPAC description

ALSPAC recruited 14,541 pregnant women resident in Avon, UK with expected dates of delivery 1st April 1991 to 31st December 1992. 14,541 is the initial number of pregnancies for which the mother enrolled in the ALSPAC study and had either returned at least one questionnaire or attended a “Children in Focus” clinic by 19/07/99. Of these initial pregnancies, there were a total of 14,676 fetuses, resulting in 14,062 live births and 13,988 children who were alive at 1 year of age.

When the oldest children were approximately 7 years of age, an attempt was made to bolster the initial sample with eligible cases who had failed to join the study originally. As a result, when considering variables collected from the age of seven onwards (and potentially abstracted from obstetric notes) there are data available for more than the 14,541 pregnancies mentioned above.

The number of new pregnancies not in the initial sample (known as Phase I enrolment) that are currently represented on the built files and reflecting enrolment status at the age of 18 is 706 (452 and 254 recruited during Phases II and III respectively), resulting in an additional 713 children being enrolled. The phases of enrolment are described in more detail in the cohort profile paper(1).

The total sample size for analyses using any data collected after the age of seven is therefore 15,247 pregnancies, resulting in 15,458 fetuses. Of this total sample of 15,458 fetuses, 14,775 were livebirths and 14,701 were alive at 1 year of age.

A 10% sample of the ALSPAC cohort, known as the Children in Focus (CiF) group, attended clinics at the University of Bristol at various time intervals between 4 to 61 months of age. The CiF group were chosen at random from the last 6 months of ALSPAC births (1,432 families attended at least one clinic). Excluded were those mothers who had moved out of the area or were lost to follow-up, and those partaking in another study of infant development in Avon.

The study website contains details of all the data that is available through a fully searchable data dictionary (<http://www.bris.ac.uk/alspac/researchers/data-access/data-dictionary/>).

## Literacy- and language-related measures in ALSPAC

*Reading accuracy and comprehension age 7 (WORD)*

Pictures and words were used to assess decoding and word reading. The child was shown a series of four pictures. Each picture had four short, simple words underneath it. The child was asked to point to the word which had the same beginning or ending sound as the picture. This was then followed by a series of three pictures, each with four words beneath, each starting with the same letter as the picture. The child was asked to point to the word that correctly named the picture. Basic reading was assessed using the basic reading subtest of the Wechsler Objective Reading Dimensions (WORD)(2). In short, the child was asked to read aloud a series of 48 unconnected words which increased in difficulty. The task was stopped after the child made six consecutive errors. The reading accuracy and comprehension score was computed as the sum of the number of items that the child read/responded to correctly.

*Reading accuracy age 9 (NBO)*

Reading accuracy was assessed by asking the child to read out ten real words selected from a larger selection of words as described by Nunes, Bryant and Olsen (NBO)(3). The test - retest reliability of word reading was 0.80. The correlation with the Schonell Word Reading Task(4) was 0.85. The reading accuracy score was computed as the sum of the number of items that the child read correctly.

*Reading speed and reading accuracy age 9 (NARA II)*

Children's reading skills were assessed with the revised Neale Analysis of Reading Ability (NARA II)(5). The child was asked to read a passage from a booklet. The tester recorded the time it took the child to read the passage, and noted any errors made by the child. All scores were standardised by age.

*Reading speed age 13 (TOWRE)*

The Test of Word Reading Efficiency (TOWRE)(6) contains a word part to assess sight word efficiency. The child had 45 seconds to read as many words as possible. Words that a child skipped, or got wrong were marked by the tester. The reading speed score was computed as the sum of the number of correct words a child finished on.

*Spelling accuracy age 7 (NB)*

Spelling accuracy was assessed by asking a child to spell a series of 15 words. The words were chosen specifically for this age group after piloting on several hundred children (Nunes and Bryant, ALSPAC-specific measure). The words included regular and irregular words of differing frequencies, and were put in an order of increasing difficulty. For each word, the tester first read the word out alone to the child, then within a specific sentence incorporating the word, and finally alone again. The child was asked to write down the spelling. The spelling accuracy score is computed as the number of words spelt correctly.

*Spelling accuracy age 9 (NB)*

The format to assess spelling accuracy at age 9 was similar to that at age 7. However, the series of 15 words that a child was asked to spell were adjusted to match the age group of 9. The spelling accuracy score is computed as the number of words spelt correctly.

*Non-word reading accuracy age 9 (NBO)*

This was assessed by asking the child to read out loud ten non-words, selected from a larger selection of non-words taken from research conducted by Nunes and colleagues(3). The test - retest reliability of the non-word reading task was 0.73. The correlation with the Schonell Word Reading Task(4) was 0.73. The tester emphasised to the child that the words ware made-up, and asked the child to read all the non-words in the way that they thought they should be read. The decoding accuracy score was computed as the sum of the number of items the child read correctly.

*Non-word reading speed age 13 (TOWRE)*

The Test of Word Reading Efficiency (TOWRE)(6) contains a non-word part to assess decoding efficiency. The child had 45 seconds to read as many non-words as possible. Words that a child skipped, or got wrong were marked by the tester. The decoding speed score is computed as the sum of the number of correct non-words a child finished on.

*Phonemic awareness age 7 (AAT)*

Phonemic awareness was assessed using the Auditory Analysis Test (AAT)(7). The task contained two practice and 40 test items of increasing difficulty. For each item, the child was first asked to repeat the word, and then produce it again but with part of the word (a phoneme or a number of phonemes) removed. There were seven omission categories included: omission of a first, medial or final syllable, omission of the initial, omission of the final consonant of a one syllable word, and omission of the first consonant or consonant blend of a medial consonant. The words from different categories were mixed. The phonemic awareness score is computed as the sum of correct responses.

*Listening comprehension age 8 (WOLD)*

A subset of the Wechsler Objective Language Dimensions (WOLD)(8) test was used to assess listening comprehension. The tester read out loud a paragraph about a picture, shown to the child. After that, the child answers ten questions on what they have heard. The listening comprehension scores were calculated as the sum of the items that the child got correct.

*Non-word repetition age 8 (CNRep)*

An adaptation of the Children’s Test of Nonword Repetition (CNRep)(9) was used to assess phonological working memory. The test comprised twelve nonsense words, four each of 3, 4 and 5 syllables. The words were conforming to English rules for sound combinations. The child was asked to listen to each word and repeat each item. If there was no phonological deviation from the target form, the repetition attempt was scored as correct. The non-word repetition score was computed as the sum of the number of correct non-words.

*Verbal intelligence age 8 (WISC-III)*

The Wechsler Intelligence Scale for Children (WISC-III)(10) was used to assess cognitive function. A short form of the measure was employed where alternate items were used for all subtests, with the exception of the coding subtest. The WISC-III comprises ten subtests five of which are verbal subtests: information, similarities, arithmetic, vocabulary, comprehension, and can be used to construct a verbal intelligence score. Raw scores were calculated according to the items used in the alternate item form of the WISC. The total age-scaled scores for the verbal scale were calculated using the look-up tables provided in the WISC manual. All scores were pro-rated.

## ALSPAC trait transformation

Literacy-related and/or language-related abilities (LRAs) were residualised for sex, age and the two most significant ancestry-informative principal components(11), and then rank-transformed unless they were derived using age-specific norms. These scores were adjusted for sex and principal components only before transformation.

## ADHD cases within ALSPAC

The Development and Wellbeing Assessment (DAWBA) was used to assess psychological disorders. It is a validated instrument combining structured and semi-structured questions related to DSM and ICD diagnostic criteria(12). DAWBA was collected through questionnaires posted to parents and teachers, and responses were reviewed by trained clinical raters who assigned diagnoses according to the DSM-IV(13). Information from both parents and teachers were combined to assign a diagnosis (similar to a clinical setting).

## ALSPAC genome-wide analyses

Standard genomic quality control including gender mismatch, heterozygosity, individual missingness, insufficient sample replication, population stratifi­cation, minor allele frequency (MAF), SNP call rate, Hardy-Weinberg equilibrium(14), and cryptic relatedness was performed using PLINK(15) (v1.07). After quality control 8 981 children and 465,740 SNPs were imputed to a HRC r1.1 reference panel(16) using the Sanger impu­tation server (EAGLE2(17) v2.0.5 and PBWT(18) software, <https://imputation.sanger.ac.uk/>). Genome-wide association analysis summary statistics for all literacy- and language-related measures were generated by regressing rank-transformed residuals on posterior genotype probabilities, assuming an additive genetic model, as implemented in SNPTEST (version 2.5.2) software(19) (without genomic control-based correction(20)).

## Clinical ADHD summary statistics

*Psychiatric Genomics Consortium (PGC)*

ADHD cases (age 5 to 17 years) met diagnostic criteria for either clinical ADHD or hyperkinetic disorder (Diagnostic and Statistical Manual of Mental Disorders (DSM-III(21), DSM-IV(13), DSM-IV-TR(22)) or the International Classification of Diseases (ICD-10(23)).

*The Lundbeck Foundation Initiative for Integrative Psychiatric Research (iPSYCH)*

ADHD cases were diagnosed according to ICD-10(23), and identified using the Danish Psychiatric Central Research Register(24). Controls were randomly selected from the same nationwide birth cohort and did not have a diagnosis of ADHD (F90.0) or moderate-severe mental retardation (F71-F79)(25,26). Genotyping was performed using the Illumina Infinium PsychArray BeadChip and genotypes were imputed to a 1000 Genomes template(27) (Phase3, release 02-05-2013). Genotyping, quality control, imputation and genetic association analysis were carried out using the Ricopili pipeline with standard PGC settings(28).

Educational attainment summary statistics

Educational attainment (EA) was coded according to the International Standard Classification of Education (1997) scale(29) and analysed as a quantitative variable defined as an individual’s years of schooling. Participants were >30 years of age at the time of assessment and of European ancestry. Genome-wide data were predominantly imputed to a 1000 genomes project(27) version 3 reference panel as described previously(30).

## Linkage Disequilibrium score regression and correlation

Linkage Disequilibrium score (LDSC) regression(31) can estimate the cumulative effect of genetic variants as tagged by common genetic markers (SNP-h^2^) to phenotypic variation, based on GWAS statistics, and distinguishes confounding from polygenic influences in genome-wide analyses^21^. To estimate LDSC-h^2^, genome-wide χ^2^-statistics are regressed on the amount of genetic variation captured by each SNP (linkage disequilibrium score, LD score)(31), while the intercept of this regression minus one is an estimator of the mean contribution of confounding bias to the inflation in the mean χ^2^-statistic^21^ . We estimated the SNP-h^2^ for LRAs and EA on the observed scale and ADHD SNP-h^2^ on the liability scale (assuming a population prevalence of 5% for ADHD(32)).

In extension, LD score correlation(33) analysis can be applied to estimate genetic correlations (r_g_) between genetic variants in distinct samples as a regression of the product of test statistics on LD score. All analyses were performed with LDSC software(31,33) and based on the set of well-imputed HapMap3 SNPs(34) and a European reference panel of LD scores(33). Unconstrained LD-score correlation(33) analysis was applied to estimate genetic correlations (r_g_) between LRAs and EA, as well as ADHD and EA, based on summary statistics from all thirteen LRAs (ALSPAC), EA (SSGAC)(30), and ADHD (PGC+iPSYCH)(25).

## Polygenic scoring analyses

Consistent with current guidelines(35), autosomal ADHD GWAS signals were clumped
(LD-r^2^>0.25, ±500 kb) with PLINK(15) software. Polygenic scores for ADHD were constructed based on *P-*value thresholds of 0.001, 0.01, 0.05, 0.10, 0.3, 0.5, 0.7, 0.9 and 1. Only imputed markers with high imputation quality (INFO(36)>0.8, 95% posterior genotyping probability >0.9 and minor allele frequency >0.005) in ALSPAC were used to generate polygenic scores.

To further illustrate the strength of the genetic overlap, we translated the fitted polygenetic model into the estimated genetic covariance. For this we used reading accuracy at age 9 (NARA II) as an example, where ADHD-PGS explained up to 1.2% of the phenotypic variation when based on the iPSYCH discovery sample. Genetic covariance was estimated from PGS results for reading accuracy at age 9 (NARA II) using Avengeme software(37). Input parameters were consistent with PGS analyses for a *P*-value threshold of 0.1, including 63,968 SNPs, a discovery sample of N=55,734, a target sample of N=5,201 and an association Z-score of -8.85 (Table S5). We furthermore assumed an ADHD prevalence of 0.05(32), a case sampling proportion of 0.36, an ADHD heritability on the liability scale of 0.21 (Table S4) and 95% null SNPs in the discovery sample.

## Overlap in genome-wide significant association signals between ADHD and educational attainment

To identify independent genetic variants passing the genome-wide significance threshold in both the ADHD (PGC+iPSYCH) and EA (SSGAC) GWASs, we first selected all SNPs with P<5x10^-8^ in either GWAS summary statistics. Next, we clumped (LD-r^2^>0.25, ±500 kb) these variants with PLINK (15) for ADHD and EA GWASs individually. Both approaches resulted in two independent overlapping variants.

## Multivariable regression

In multivariable regression (MVR) analyses, genetic effects for the outcome are regressed on the genetic effects for risk factor and covariates, using a multivariable weighted regression model(38). The model allows for association between risk factor and outcome (risk-factor specific associations), but also covariate and outcome, based on the same set of instruments, where latter capture pleiotropic effects. However, the model does not imply causal inferences as modelling assumptions are not met.

Using effect estimates from GWAS summary statistics ($\hat{\beta}$), the specific effect of a risk factor
($\theta_{s}$) conditional on a covariate ($\theta_{C}$) on the outcome can be estimated using the weighted regression:. For a valid estimation of effects the following assumptions need to be met: 1) the genetic variants are associated with the risk factor and covariate, 2) the genetic variants are not associated with confounders, and 3) there is no pathway from any genetic variant to the outcome except via the risk factor and/or confounder(38).

In order to investigate whether the third assumption holds we investigated the evidence for a regression intercept in an unconstrained regression model. An intercept consistent with zero (i.e. within the 95% confidence interval) suggests that there is no evidence for additional pleiotropic effects. Since the intercept is, however, sensitive to the defined direction of effect, unconstrained models were fitted twice, once with genetic variants aligned according to the risk factor and once according to the covariate.

## Meta-regression across literacy- and language-related abilities

The genetic and phenotypic inter-relatedness among LRAs needs to be accounted for when combining ADHD effects (shared with and independent of EA) across multiple LRA combinations. A variance/covariance matrix across the correlated LRAs was approximated analogous to models accounting for correlated phylogenetic histories(39). The matrix was based on the observed phenotypic correlation matrix using rank-transformed measures (Table S1) and weighted by the standard errors of the estimated polygenic association. The model included, for each LRA combination, one random intercept. Evidence for polygenic effect heterogeneity was assessed using Cochran's Q-test.

MVR estimates for defined LRA combinations were contrasted with each other by conducting moderator analyses as part of a random-effects meta-regression model (R:metafor library(40), Rv3.2.0) across the entire set of LRAs studied (at a significance level of 0.05). This includes a dummy coded contrast, the moderator (mod), that is added to the model in order to explain heterogeneity in effect estimates, and an assessment of the remaining residual effect heterogeneity (res het). Note that verbal intelligence quotient scores were excluded from the analysis, as they represent a composite measure.

## Standardisation of genetic effect estimates

To compare the magnitude of regression estimates capturing polygenic ADHD effects on LRAs independent of and shared with EA, we calculated standardised SNP effects for ADHD (PGC+iPSYCH), EA (SSGAC) and LRAs (ALSPAC) from GWAS summary statistics.

Standardised regression coefficients for SNP j with minor allele frequency MAF_j_ and sample size N_j_ were calculated as(29)

$\hat{\beta}_{j}=\frac{z_{j}\times\hat{\sigma_{y}}}{\sqrt{N_{j}\times\sigma_{x_{j}}^{2}}}$ $\hat{\beta}_{j}=\frac{z_{j}\times\hat{\sigma_{y}}}{\sqrt{\hat{\beta}_{j}=\frac{z_{j}\times\hat{\sigma_{y}}}{\sqrt{\hat{\beta}_{j}=\frac{z_{j}\times\hat{\sigma_{y}}}{\sqrt{N_{j}\times\sigma_{x_{j}}^{2}}}N_{j}\times\sigma_{x_{j}}^{2}}}N_{j}\times\hat{\sigma}_{x_{j}}^{2}}}$ $\hat{\beta}_{j}=\frac{z_{j}\times\hat{\sigma_{y}}}{\sqrt{N_{j}\times\hat{\sigma}_{x_{j}}^{2}}}$ (equation 1) where $z_{j}$ is the Wald test statistic, $\hat{\sigma}_{y}$ is the standard deviation of the phenotype y, and $\hat{\sigma}_{x_{j}}^{2}$ corresponds to the SNP variance 2 x MAF_j_ x (1-MAF_j_). The corresponding standard error was calculated as(29)

(equation 2)

We estimated $\hat{\sigma}_{y}$ for each component of the multivariable regression model as follows.

*Literacy- and language-related abilities*

All population-based linguistic traits were rank-transformed (continuous traits) and thus $\hat{\sigma}_{y}$ equals to one.

*Educational attainment*

The combined $\hat{\sigma}_{y}$ for EA, measured as continuous score in years of schooling, was pooled from 63 cohorts(30,41)

(equation 3)

with sample size$n_{i}$ , sample variance $\sigma_{i}^{2}$, and number of studies $k$.

*ADHD*

The estimated standard deviation of the liability of ADHD $\hat{\sigma}_{y*}$ , taking the prevalence of the disorder $K$ (0.05 for ADHD(32)) and sample prevalence $P$ (0.364 for PGC+iPSYCH ADHD(25)) into account, was calculated as(42)

$\hat{\sigma}_{y*}=\sqrt{1+\frac{z_{trans}(P-K)}{K(1-K)}\cdot\left(t-\frac{z_{trans}(P-K)}{K(1-K)}\right)}$ (equation 4)

where $t$ is the truncation threshold (derived as the inverse standard normal distribution function at $1-K$) and $z_{trans}$ the height of the standard normal probability density function at$t$.

## Attrition analysis in ALSPAC

A dichotomous variable indicating missingness (coded as 1) for reading accuracy and comprehension at age 7 (WORD) was created for each child that was alive at the age of one year and had genotype data available (N_total_=8,095; N_missing_=2,204). Genome-wide association analysis summary statistics were generated by logistic regression of sample drop-out on posterior genotype probabilities, assuming an additive genetic model, as implemented in SNPTEST (version 2.5.2) software(19) (without genomic control-based correction(20)).

ADHD polygenic scores were regressed on data missingness for reading accuracy and comprehension at age 7 (WORD) using logistic regression (R:stats library, Rv3.2.3) to estimate odds ratios and their standard errors per one-standard-deviation increase in polygenic scores (Methods). McFadden's pseudo-R^2^ values for logistic regression were estimated (R:pscl library, Rv3.2.3), analogous to the ordinary least square regression R^2^.

Utilising a multivariable regression approach(38) (see Methods), analysis were subsequently carried out conditional on genetically predicted educational attainment (EA), a potential correlate for non-participation(43), using GWAS summary statistics. We dissected ADHD polygenic influences on LRA-missingness into genetic effects that are independent of EA and genetic effects that are shared with EA, using both conservative (*P*-threshold *P*<5x10^-8^) and subthreshold ADHD variants (*P*-threshold *P*<0.0015), as described for multivariable regression analyses (see Methods).

We identified a positive genetic association between sample-dropout and ADHD-PGS (PGC+iPSYCH, OR=1.03(SE=0.005), *P*=1.4x10^-8^, Table S12), consistent with previous studies(44). Disentangling this polygenic link using MVR (Table S13) based on ADHD subthreshold instruments showed a 0.05 increase in log-odds sample drop-out per log-odds increase in liability to ADHD, independent of EA (log(OR)=0.05(SE=0.01), *P*=3.7x10^-4^). Additionally, a further 1.04 increase in log odds sample drop-out per missing year of schooling (log(OR)=1.04(SE=0.19),*P*=7.3x10^-8^) was observed, suggesting also ADHD effects shared with EA.

## Web resources

ALSPAC data dictionary: <http://www.bris.ac.uk/alspac/researchers/data-access/data-dictionary/>

ALSPAC variable catalogue: <http://www.bristol.ac.uk/alspac/researchers/access/>

PGC: <http://www.med.unc.edu/pgc>

PLINK: <https://www.cog-genomics.org/plink2>

HRC: <http://www.haplotype-reference-consortium.org/>

SANGER IMPUTATION SERVER: <https://imputation.sanger.ac.uk/>

SNPTEST: <https://mathgen.stats.ox.ac.uk/genetics_software/snptest/snptest.html>

LDSC: <https://github.com/bulik/ldsc>

GCTA: <http://cnsgenomics.com/software/gcta/#Overview>

GCTA power: <http://cnsgenomics.com/shiny/gctaPower/>

R: <https://www.r-project.org/>

METAFOR: <http://www.metafor-project.org/doku.php>

# Supplementary References

1. Boyd A, Golding J, Macleod J, Lawlor DA, Fraser A, Henderson J, et al. Cohort Profile: the ’children of the 90s’--the index offspring of the Avon Longitudinal Study of Parents and Children. Int J Epidemiol. 2013 Feb;42(1):111–27.

2. WORD, Wechsler Objective Reading Dimensions Manual. Psychological Corporation; 1993. 146 p.

3. Nunes T, Bryant P, Olsson J. Learning Morphological and Phonological Spelling Rules: An Intervention Study. Sci Stud Read. 2003 Jul;7(3):289–307.

4. Schonell FJ, Goodacre E. The Psychology and Teaching of Reading. Fifth Edition. 1974;

5. Neale MD. Neale Analysis of Reading Ability: Second Revised British Edition. London: NFER-Nelson; 1997.

6. TOWRE, Test of Word Reading Efficiency: Examiner’s Manual. PRO-ED; 1999. 108 p.

7. Rosner J, Simon DP. The Auditory Analysis Test: An Initial Report. J Learn Disabil. 1971 Aug 1;4(7):384–92.

8. Rust J, Wechsler D. WOLD: Wechsler objective language dimensions. London; [Hong Kong: Psychological Corp. ; Artsberg Enterprises Ltd. [distributor; 1996.

9. Gathercole SE, Willis CS, Baddeley AD, Emslie H. The Children’s Test of Nonword Repetition: a test of phonological working memory. Mem Hove Engl. 1994 Jun;2(2):103–27.

10. Wechsler D, Golombok S, Rust J. WISC-III UK Wechsler Intelligence Scale for Children – Third Edition UK Manual. Sidcup, UK: The Psychological Corporation; 1992.

11. Price AL, Patterson NJ, Plenge RM, Weinblatt ME, Shadick NA, Reich D. Principal components analysis corrects for stratification in genome-wide association studies. Nat Genet. 2006 Aug;38(8):904–9.

12. Goodman R, Ford T, Richards H, Gatward R, Meltzer H. The Development and Well-Being Assessment: Description and Initial Validation of an Integrated Assessment of Child and Adolescent Psychopathology. J Child Psychol Psychiatry. 2000 Jul 1;41(5):645–55.

13. American Psychiatric Association. Diagnostic and statistical manual of mental disorders. Vol. fourth. Washington, DC: American Psychiatric Association;

14. Wigginton JE, Cutler DJ, Abecasis GR. A Note on Exact Tests of Hardy-Weinberg Equilibrium. Am J Hum Genet. 2005 May;76(5):887–93.

15. Purcell S, Neale B, Todd-Brown K, Thomas L, Ferreira MAR, Bender D, et al. PLINK: A Tool Set for Whole-Genome Association and Population-Based Linkage Analyses. Am J Hum Genet. 2007 Sep;81(3):559–75.

16. McCarthy S, Das S, Kretzschmar W, Delaneau O, Wood AR, Teumer A, et al. A reference panel of 64,976 haplotypes for genotype imputation. Nat Genet. 2016 Oct;48(10):1279–83.

17. Loh P-R, Danecek P, Palamara PF, Fuchsberger C, A Reshef Y, K Finucane H, et al. Reference-based phasing using the Haplotype Reference Consortium panel. Nat Genet. 2016 Nov;48(11):1443–8.

18. Durbin R. Efficient haplotype matching and storage using the positional Burrows-Wheeler transform (PBWT). Bioinforma Oxf Engl. 2014 May 1;30(9):1266–72.

19. Marchini J, Howie B, Myers S, McVean G, Donnelly P. A new multipoint method for genome-wide association studies by imputation of genotypes. Nat Genet. 2007 Jul;39(7):906–13.

20. Devlin B, Roeder K. Genomic control for association studies. Biometrics. 1999 Dec;55(4):997–1004.

21. American Psychiatric Association. Diagnostic and statistical manual of mental disorders. Vol. third. Washington, DC: American Psychiatric Association; 1980.

22. American Psychiatric Association. Diagnostic and statistical manual of mental disorders. Vol. fourth, text revision. Washington, DC: American Psychiatric Association;

23. International Statistical Classification of Diseases and Related Health Problems. Vol. 10th revision. Malta: World Health Organization; 2010.

24. Mors O, Perto GP, Mortensen PB. The Danish Psychiatric Central Research Register. Scand J Public Health. 2011 Jul;39(7 Suppl):54–7.

25. Demontis D, Walters RK, Martin J, Mattheisen M, Als TD, Agerbo E, et al. Discovery Of The First Genome-Wide Significant Risk Loci For ADHD. bioRxiv. 2017 Jun 3;145581.

26. Pedersen CB, Bybjerg-Grauholm J, Pedersen MG, Grove J, Agerbo E, Bækvad-Hansen M, et al. The iPSYCH2012 case-cohort sample: new directions for unravelling genetic and environmental architectures of severe mental disorders. Mol Psychiatry. 2017 Sep 19;

27. The 1000 Genomes Project Consortium. A global reference for human genetic variation. Nature. 2015 Oct 1;526(7571):68–74.

28. Schizophrenia Working Group of the Psychiatric Genomics Consortium. Biological insights from 108 schizophrenia-associated genetic loci. Nature. 2014 Jul 24;511(7510):421–7.

29. Rietveld CA, Medland SE, Derringer J, Yang J, Esko T, Martin NW, et al. GWAS of 126,559 individuals identifies genetic variants associated with educational attainment. Science. 2013 Jun 21;340(6139):1467–71.

30. Okbay A, Beauchamp JP, Fontana MA, Lee JJ, Pers TH, Rietveld CA, et al. Genome-wide association study identifies 74 loci associated with educational attainment. Nature. 2016 May 26;533(7604):539–42.

31. Bulik-Sullivan BK, Loh P-R, Finucane HK, Ripke S, Yang J, Schizophrenia Working Group of the Psychiatric Genomics Consortium, et al. LD Score regression distinguishes confounding from polygenicity in genome-wide association studies. Nat Genet. 2015 Mar;47(3):291–5.

32. Polanczyk G, de Lima MS, Horta BL, Biederman J, Rohde LA. The Worldwide Prevalence of ADHD: A Systematic Review and Metaregression Analysis. Am J Psychiatry. 2007 Jun 1;164(6):942–8.

33. Bulik-Sullivan B, Finucane HK, Anttila V, Gusev A, Day FR, Loh P-R, et al. An atlas of genetic correlations across human diseases and traits. Nat Genet. 2015 Sep 28;47:1236–1241.

34. International HapMap 3 Consortium. Integrating common and rare genetic variation in diverse human populations. Nature. 2010 Sep 2;467(7311):52–8.

35. Wray NR, Lee SH, Mehta D, Vinkhuyzen AAE, Dudbridge F, Middeldorp CM. Research Review: Polygenic methods and their application to psychiatric traits. J Child Psychol Psychiatry. 2014 Oct 1;55(10):1068–87.

36. Howie BN, Donnelly P, Marchini J. A Flexible and Accurate Genotype Imputation Method for the Next Generation of Genome-Wide Association Studies. PLOS Genet. 2009 Jun 19;5(6):e1000529.

37. Palla L, Dudbridge F. A Fast Method that Uses Polygenic Scores to Estimate the Variance Explained by Genome-wide Marker Panels and the Proportion of Variants Affecting a Trait. Am J Hum Genet. 2015 Aug 6;97(2):250–9.

38. Burgess S, Thompson DJ, Rees JMB, Day FR, Perry JR, Ong KK. Dissecting Causal Pathways Using Mendelian Randomization with Summarized Genetic Data: Application to Age at Menarche and Risk of Breast Cancer. Genetics. 2017 Jan 1;genetics.300191.2017.

39. Lajeunesse MJ. Meta‐Analysis and the Comparative Phylogenetic Method. Am Nat. 2009 Sep;174(3):369–81.

40. Viechtbauer W. Conducting meta-analyses in R with the metafor package. J Stat Softw. 2010;1–48.

41. Pooled Variance. In: The Concise Encyclopedia of Statistics [Internet]. Springer New York; 2008 [cited 2017 Aug 22]. p. 427–8. Available from: http://link.springer.com/referenceworkentry/10.1007/978-0-387-32833-1_323

42. Lee SH, Wray NR, Goddard ME, Visscher PM. Estimating Missing Heritability for Disease from Genome-wide Association Studies. Am J Hum Genet. 2011 Mar 11;88(3):294–305.

43. Martin J, Tilling K, Hubbard L, Stergiakouli E, Thapar A, Smith GD, et al. Association of Genetic Risk for Schizophrenia With Nonparticipation Over Time in a Population-Based Cohort Study. Am J Epidemiol. 2016 May 10;kww009.

44. Taylor A, Jones H, Sallis H, Euesden J, Stergiakouli E, Davies N, et al. The molecular genetics of participation in the Avon Longitudinal Study of Parents and Children. bioRxiv. 2017 Oct 20;206698.

45. Yang J, Benyamin B, McEvoy BP, Gordon S, Henders AK, Nyholt DR, et al. Common SNPs explain a large proportion of the heritability for human height. Nat Genet. 2010 Jul;42(7):565–9.

# Supplementary Tables

## Table S1: Phenotypic correlations among literacy- and language-related measures

|  | Reading a/c 7 (WORD) | Reading a 9 (NBO) | Reading s 9 (NARA II) | Reading a 9 (NARA II) | Reading s 13 (TOWRE) | NW reading a 9 (NBO) | NW reading s 13 (TOWRE) | Spelling a 7 (NB) | Spelling a 9 (NB) | PhonAware 7 (AAT) | Listening c 8 (WOLD) | Non-word repetition 8 (CNRep) | VIQ 8 (WISC-III) |
| --- | --- | --- | --- | --- | --- | --- | --- | --- | --- | --- | --- | --- | --- |
| Reading a/c 7 (WORD) | 1 | 0.72 | 0.71 | 0.83 | 0.54 | 0.66 | 0.59 | 0.84 | 0.75 | 0.70 | 0.27 | 0.42 | 0.55 |
| Reading a 9 (NBO) | 0.72 | 1 | 0.63 | 0.76 | 0.50 | 0.72 | 0.56 | 0.67 | 0.71 | 0.57 | 0.23 | 0.36 | 0.45 |
| Reading s 9 (NARA II) | 0.71 | 0.63 | 1 | 0.74 | 0.62 | 0.56 | 0.65 | 0.63 | 0.65 | 0.48 | 0.30 | 0.34 | 0.50 |
| Reading a 9 (NARA II) | 0.82 | 0.77 | 0.74 | 1 | 0.59 | 0.70 | 0.65 | 0.75 | 0.78 | 0.64 | 0.29 | 0.44 | 0.55 |
| Reading s 13 (TOWRE) | 0.53 | 0.50 | 0.62 | 0.60 | 1 | 0.45 | 0.81 | 0.48 | 0.52 | 0.41 | 0.24 | 0.29 | 0.42 |
| NW reading a 9 (NBO) | 0.66 | 0.72 | 0.56 | 0.70 | 0.45 | 1 | 0.53 | 0.63 | 0.66 | 0.55 | 0.17 | 0.33 | 0.39 |
| NW reading s 13 (TOWRE) | 0.58 | 0.57 | 0.65 | 0.65 | 0.81 | 0.53 | 1 | 0.54 | 0.58 | 0.45 | 0.20 | 0.28 | 0.38 |
| Spelling a 7 (NB) | 0.84 | 0.66 | 0.62 | 0.74 | 0.47 | 0.62 | 0.53 | 1 | 0.77 | 0.66 | 0.19 | 0.35 | 0.46 |
| Spelling a 9 (NB) | 0.74 | 0.71 | 0.65 | 0.78 | 0.51 | 0.66 | 0.58 | 0.75 | 1 | 0.59 | 0.21 | 0.36 | 0.47 |
| PhonAware 7 (AAT) | 0.70 | 0.57 | 0.48 | 0.64 | 0.40 | 0.54 | 0.45 | 0.66 | 0.58 | 1 | 0.19 | 0.38 | 0.45 |
| Listening c 8 (WOLD) | 0.27 | 0.23 | 0.31 | 0.29 | 0.24 | 0.17 | 0.20 | 0.20 | 0.20 | 0.19 | 1 | 0.23 | 0.44 |
| Non-word repetition 8 (CNRep) | 0.42 | 0.36 | 0.34 | 0.43 | 0.29 | 0.32 | 0.28 | 0.35 | 0.36 | 0.38 | 0.23 | 1 | 0.37 |
| VIQ 8 (WISC-III) | 0.54 | 0.45 | 0.51 | 0.56 | 0.42 | 0.39 | 0.39 | 0.45 | 0.47 | 0.45 | 0.43 | 0.37 | 1 |

Abbreviations: a, accuracy; c, comprehension; s, speed; WORD, Wechsler Objective Reading Dimension; NBO, Nunes, Bryant and Olson (ALSPAC specific instrument); NARA II, The Neale Analysis of Reading Ability- Second Revised British Edition; TOWRE, Test Of Word Reading Efficiency; NW, non-word; NB, Nunes and Bryant (ALSPAC specific instrument); PhonAware, phonemic awareness, AAT, Auditory Analysis Test; WOLD, Wechsler Objective Language Dimensions; CNRep, Children’s Test of Nonword Repetition; VIQ, verbal intelligence quotient; WISC-III, Wechsler Intelligence Scale for Children III

Phenotypic correlations are depicted as Pearson's correlation coefficients. The upper triangle represents phenotypic correlations based on rank-transformed measures, whereas the lower triangle represents phenotypic correlations based on untransformed measures.

##

## Table S2: SNP-heritability estimates for literacy- and language-related measures

| **LRAs** | **LDSC** | | | | | **REML** | |
| --- | --- | --- | --- | --- | --- | --- | --- |
|  | **SNP-h^2^ (SE)** | **Mean**  **chi-square** | **N** | **λ_GC_** | **Intercept (SE)** | **SNP-h^2^ (SE)** | **N^1^** |
| Reading a/c 7 (WORD) | 0.35 (0.08) | 1.05 | 5 891 | 1.05 | 1.01 (0.01) | 0.42 (0.06) | 5 723 |
| Reading a 9 (NBO) | 0.31 (0.09) | 1.05 | 5 738 | 1.05 | 1.01 (0.01) | 0.46 (0.06) | 5 574 |
| Reading s 9 (NARA II) | 0.32 (0.10) | 1.05 | 5 189 | 1.03 | 1.01 (0.01) | 0.45 (0.07) | 5 037 |
| Reading a 9 (NARA II) | 0.43 (0.10) | 1.05 | 5 201 | 1.05 | 1.01 (0.01) | 0.50 (0.07) | 5 048 |
| Reading s 13 (TOWRE) | 0.24 (0.11) | 1.03 | 4 247 | 1.03 | 1.01 (0.01) | 0.40 (0.09) | 4 131 |
| NW reading a 9 (NBO) | 0.27 (0.09) | 1.04 | 5 731 | 1.04 | 1.01 (0.01) | 0.32 (0.06) | 5 569 |
| NW reading s 13 (TOWRE) | 0.18 (0.11) | 1.03 | 4 237 | 1.03 | 1.01 (0.01) | 0.38 (0.09) | 4 121 |
| Spelling a 7 (NB) | 0.29 (0.08) | 1.04 | 5 800 | 1.04 | 1.00 (0.01) | 0.32 (0.06) | 5 637 |
| Spelling a 9 (NB) | 0.25 (0.09) | 1.04 | 5 728 | 1.04 | 1.01 (0.01) | 0.38 (0.06) | 5 564 |
| PhonAware 7 (AAT) | 0.34 (0.08) | 1.05 | 5 919 | 1.04 | 1.00 (0.01) | 0.39 (0.06) | 5 749 |
| Listening c 8 (WOLD) | 0.23 (0.08) | 1.03 | 5 473 | 1.03 | 1.01 (0.01) | 0.32 (0.07) | 5 324 |
| Non-word repetition 8 (CNRep) | 0.14 (0.09) | 1.03 | 5 464 | 1.04 | 1.01 (0.01) | 0.32 (0.07) | 5 315 |
| VIQ 8 (WISC-III) | 0.42 (0.10) | 1.06 | 5 456 | 1.06 | 1.02 (0.01) | 0.54 (0.07) | 5 305 |

1. Differences compared with the total sample N are due to excluding individuals with a genetic relationship of ≥0.05(45)

Abbreviations: LRAs, literacy and language-related abilities; LDSC, LD score regression; REML, Restricted Maximum Likelihood (REML) analyses as implemented in genome-wide complex trait analysis software; N, sample size; λ_GC_, lambda GC; a, accuracy; c, comprehension; s, speed; WORD, Wechsler Objective Reading Dimension; NBO, Nunes, Bryant and Olson (ALSPAC specific instrument); NARA II, The Neale Analysis of Reading Ability- Second Revised British Edition; TOWRE, Test Of Word Reading Efficiency; NW, non-word; NB, Nunes and Bryant (ALSPAC specific instrument); PhonAware, phonemic awareness; AAT, Auditory Analysis Test; WOLD, Wechsler Objective Language Dimensions; CNRep, Children’s Test of Nonword Repetition; VIQ, verbal intelligence quotient; WISC-III, Wechsler Intelligence Scale for Children III

##

## Table S3: Genetic correlations among literacy- and language-related measures

|  | Reading a/c 7 (WORD) | Reading a 9 (NBO) | Reading s 9 (NARA II) | Reading a 9 (NARA II) | Reading s 13 (TOWRE) | NW reading a 9 (NBO) | NW reading s (TOWRE) | Spelling a 7 (NB) | Spelling a 9 (NB) | PhonAware 7 (AAT) | Listening c 8 (WOLD) | Non-word repetition 8 (CNRep) | VIQ 8 (WISC-III) |
| --- | --- | --- | --- | --- | --- | --- | --- | --- | --- | --- | --- | --- | --- |
| Reading a/c 7 (WORD) | 1 |
| Reading a 9 (NBO) | 0.88 (0.04) | 1 |
| Reading s 9 (NARA II) | 0.97 (0.05) | 0.84 (0.06) | 1 |
| Reading a 9 (NARA II) | 1.00 (0.03) | 0.89 (0.04) | 0.93 (0.04) | 1 |
| Reading s 13 (TOWRE) | 0.81 (0.09) | 0.67 (0.09) | 0.94 (0.08) | 0.90 (0.08) | 1 |
| NW reading a 9 (NBO) | 0.92 (0.06) | 0.98 (0.05) | 0.81 (0.08) | 0.93 (0.05) | 0.68 (0.11) | 1 |
| NW reading s (TOWRE) | 0.88 (0.08) | 0.92 (0.08) | 0.92 (0.07) | 0.94 (0.07) | 0.87 (0.04) | 0.96 (0.11) | 1 |
| Spelling a 7 (NB) | 0.96 (0.03) | 0.87 (0.06) | 0.82 (0.07) | 0.96 (0.05) | 0.71 (0.11) | 0.93 (0.08) | 0.86 (0.11) | 1 |
| Spelling a 9 (NB) | 0.94 (0.04) | 0.95 (0.04) | 0.90 (0.06) | 0.96 (0.03) | 0.83 (0.09) | 0.46 (0.14) | 0.49 (0.15) | 0.97 (0.04) | 1 |
| PhonAware 7 (AAT) | 0.97 (0.04) | 0.86 (0.07) | 0.86 (0.09) | 0.97 (0.06) | 0.77 (0.12) | 0.93 (0.08) | 0.86 (0.11) | 0.98 (0.05) | 0.87 (0.10) | 1 |
| Listening c 8 (WOLD) | 0.74 (0.11) | 0.49 (0.12) | 0.80 (0.12) | 0.67 (0.11) | 0.58 (0.14) | 0.46 (0.14) | 0.49 (0.15) | 0.73 (0.14) | 0.55 (0.13) | 0.64 (0.13) | 1 |
| Non-word repetition 8 (CNRep) | 0.68 (0.10) | 0.61 (0.61) | 0.69 (0.12) | 0.73 (0.10) | 0.41 (0.14) | 0.72 (0.12) | 0.40 (0.15) | 0.66 (0.12) | 0.81 (0.11) | 0.70 (0.11) | 0.54 (0.14) | 1 |
| VIQ 8 (WISC-III) | 0.91 (0.06) | 0.66 (0.07) | 0.86 (0.07) | 0.84 (0.06) | 0.80 (0.10) | 0.68 (0.09) | 0.74 (0.10) | 0.81 (0.08) | 0.72 (0.08) | 0.77 (0.07) | 0.88 (0.09) | 0.71 (0.09) | 1 |

Abbreviations: a, accuracy; c, comprehension; s, speed; WORD, Wechsler Objective Reading Dimension; NBO, Nunes, Bryant and Olson (ALSPAC specific instrument); NARA II, The Neale Analysis of Reading Ability- Second Revised British Edition; TOWRE, Test Of Word Reading Efficiency; NW, non-word; NB, Nunes and Bryant (ALSPAC specific instrument); PhonAware, phonemic awareness; AAT, Auditory Analysis Test; WOLD, Wechsler Objective Language Dimensions; CNRep, Non-Word Repetition test; VIQ, verbal intelligence quotient; WISC-III, Wechsler Intelligence Scale for Children III

Genetic correlations were calculated based on rank-transfromed scores using Restricted Maximum Likelihood (REML) analyses as implemented in genome-wide complex trait analysis software, based on samples of individuals with a genetic relationship of <0.05. Standard errors are provided in brackets.

## Table S4: SNP-heritability estimates for clinical ADHD and educational attainment

| **Phenotype** | **Sample** | **SNP-h^2^ (SE)** | **λ_GC_** | **Intercept (SE)** | |  |
| --- | --- | --- | --- | --- | --- | --- |
| ADHD | PGC | 0.08 (0.03) | 1.00 | | 0.99 (0.01) | |
|  | iPSYCH | 0.26 (0.02) | 1.23 | | 1.03 (0.01)^b^ | |
|  | PGC+iPSYCH (EUR) | 0.22 (0.01) | 1.25 | | 1.04 (0.01)^b^ | |
|  | PGC+iPSYCH | 0.21 (0.01) | 1.25 | | 1.04 (0.01)^b^ | |
| EA | SSGAC | 0.11 (0.004) | 1.47 | | 0.96 (0.01) | |

Abbreviations: ADHD, Attention-Deficit/Hyperactivity Disorder; EA, educational attainment; λ_GC_, lambda GC; PGC, Psychiatric Genomics Consortium; iPSYCH, The Lundbeck Foundation Initiative for Integrative Psychiatric Research; EUR, European ancestry; SSGAC, Social Science Genetic Association Consortium.

SNP-heritability was estimated with LDSC regression analysis. SNP-heritability estimates for ADHD samples were calculated on a liability scale assuming a population prevalence of 0.05(32).

## Table S5: Association of polygenic ADHD risk scores with literacy- and language-related measures

| **LRAs** | **ADHD sample** | **β (SE)** | ***P*** | **R^2^ (%)** |
| --- | --- | --- | --- | --- |
| Reading a/c 7 (WORD) | PGC | -0.04 (0.01) | 4.6x10^-3^ | 0.14 |
|  | iPSYCH | -0.10 (0.01) | <1x10^-10^ | 0.99 |
|  | PGC+iPSYCH | -0.11 (0.01) | <1x10^-10^ | 1.33 |
|  | PGC+iPSYCH(EUR) | -0.12 (0.01) | <1x10^-10^ | 1.42 |
| Reading a 9 (NBO) | PGC | -0.02 (0.01) | 6.4x10^-2^ | 0.06 |
|  | iPSYCH | -0.09 (0.01) | <1x10^-10^ | 0.73 |
|  | PGC+iPSYCH | -0.10 (0.01) | <1x10^-10^ | 0.93 |
|  | PGC+iPSYCH(EUR) | -0.10 (0.01) | <1x10^-10^ | 0.92 |
| Reading s 9 (NARA II) | PGC | -0.04 (0.01) | 7.6x10^-3^ | 0.14 |
|  | iPSYCH | -0.10 (0.01) | <1x10^-10^ | 1.02 |
|  | PGC+iPSYCH | -0.11 (0.01) | <1x10^-10^ | 1.18 |
|  | PGC+iPSYCH(EUR) | -0.11 (0.01) | <1x10^-10^ | 1.28 |
| Reading a 9 (NARA II) | PGC | -0.04 (0.01) | 5.7x10^-3^ | 0.15 |
|  | iPSYCH | -0.11 (0.01) | <1x10^-10^ | 1.20 |
|  | PGC+iPSYCH | -0.12 (0.01) | <1x10^-10^ | 1.49 |
|  | PGC+iPSYCH(EUR) | -0.12 (0.01) | <1x10^-10^ | 1.47 |
| Reading s 13 (TOWRE) | PGC | -0.03 (0.02) | 2.5x10^-2^ | 0.12 |
|  | iPSYCH | -0.09 (0.02) | 4.2x10^-9^ | 0.81 |
|  | PGC+iPSYCH | -0.11 (0.02) | <1x10^-10^ | 1.19 |
|  | PGC+iPSYCH(EUR) | -0.11 (0.02) | <1x10^-10^ | 1.22 |
| NW reading a 9 (NBO) | PGC | -0.03 (0.01) | 4.8x10^-2^ | 0.07 |
|  | iPSYCH | -0.07 (0.01) | 4.7x10^-8^ | 0.52 |
|  | PGC+iPSYCH | -0.09 (0.01) | <1x10^-10^ | 0.76 |
|  | PGC+iPSYCH(EUR) | -0.09 (0.01) | <1x10^-10^ | 0.77 |
| NW reading s 13 (TOWRE) | PGC | -0.03 (0.02) | 4.7x10^-2^ | 0.09 |
|  | iPSYCH | -0.09 (0.02) | 2.6x10^-8^ | 0.73 |
|  | PGC+iPSYCH | -0.11 (0.02) | <1x10^-10^ | 1.14 |
|  | PGC+iPSYCH(EUR) | -0.10 (0.02) | <1x10^-10^ | 1.08 |
| Spelling a 7 (NB) | PGC | -0.03 (0.01) | 9.6x10^-3^ | 0.12 |
|  | iPSYCH | -0.09 (0.01) | <1x10^-10^ | 0.86 |
|  | PGC+iPSYCH | -0.11 (0.01) | <1x10^-10^ | 1.17 |
|  | PGC+iPSYCH(EUR) | -0.11 (0.01) | <1x10^-10^ | 1.16 |
| Spelling a 9 (NB) | PGC | -0.04 (0.01) | 1.5x10^-3^ | 0.18 |
|  | iPSYCH | -0.09 (0.01) | <1x10^-10^ | 0.81 |
|  | PGC+iPSYCH | -0.10 (0.01) | <1x10^-10^ | 1.08 |
|  | PGC+iPSYCH(EUR) | -0.10 (0.01) | <1x10^-10^ | 1.06 |
| PhonAware 7 (AAT) | PGC | -0.02 (0.01) | 2.4x10^-1^ | 0.02 |
|  | iPSYCH | -0.08 (0.01) | 2.9x10^-9^ | 0.59 |
|  | PGC+iPSYCH | -0.09 (0.01) | <1x10^-10^ | 0.84 |
|  | PGC+iPSYCH(EUR) | -0.10 (0.01) | <1x10^-10^ | 0.98 |
| Listening c 8 (WOLD) | PGC | -0.02 (0.01) | 1.9x10^-1^ | 0.03 |
|  | iPSYCH | -0.06 (0.01) | 9.9x10^-6^ | 0.36 |
|  | PGC+iPSYCH | -0.08 (0.01) | 2.5x10^-8^ | 0.57 |
|  | PGC+iPSYCH(EUR) | -0.08 (0.01) | 9.2x10^-10^ | 0.68 |
| Non-word repetition 8 (CNRep) | PGC | -0.003 (0.01) | 8.1x10^-1^ | 0.001 |
|  | iPSYCH | -0.05 (0.01) | 1.7x10^-4^ | 0.26 |
|  | PGC+iPSYCH | -0.06 (0.01) | 6.3x10^-6^ | 0.37 |
|  | PGC+iPSYCH(EUR) | -0.07 (0.01) | 2.3x10^-7^ | 0.49 |
| VIQ 8 (WISC-III) | PGC | -0.03 (0.01) | 2.8x10^-2^ | 0.09 |
|  | iPSYCH | -0.11 (0.01) | <1x10^-10^ | 1.28 |
|  | PGC+iPSYCH | -0.13 (0.01) | <1x10^-10^ | 1.59 |
|  | PGC+iPSYCH(EUR) | -0.13 (0.01) | <1x10^-10^ | 1.69 |

Abbreviations: LRAs, literacy- and language-related abilities; ADHD, Attention-Deficit/Hyperactivity Disorder; R^2^, OLS-regression R^2^; a, accuracy; c, comprehension; s, speed; WORD, Wechsler Objective Reading Dimension; NBO, Nunes, Bryant and Olson (ALSPAC specific instrument); NARA II, The Neale Analysis of Reading Ability- Second Revised British Edition; TOWRE, Test Of Word Reading Efficiency; NW, non-word; NB, Nunes and Bryant (ALSPAC specific instrument); PhonAware, phonemic awareness; AAT, Auditory Analysis Test; WOLD, Wechsler Objective Language Dimensions; CNRep, Children’s Test of Nonword Repetition; VIQ, verbal intelligence quotient; WISC-III, Wechsler Intelligence Scale for Children III; PGC, Psychiatric Genomics Consortium; iPSYCH, The Lundbeck Foundation Initiative for Integrative Psychiatric Research; EUR, European descent.

ADHD SNPs were selected from GWAS summary statistics based on a *P*-value threshold of 0.1, and alleles were aligned such that the effect allele increased ADHD risk. LRAs were regressed on polygenic ADHD risk scores using ordinary least square regression. Effects were considered as significant if they passed the experiment-wide significance threshold (*P*<0.007).

## Table S6: Association of polygenic ADHD risk scores with literacy- and language-related measures excluding ADHD children

| **LRAs** | **ADHD sample** | **β (SE)** | ***P*** | **R^2^ (%)** |
| --- | --- | --- | --- | --- |
| Reading a/c 7 (WORD) | PGC | -0.04 (0.01) | 3.5x10^-3^ | 0.15 |
|  | iPSYCH | -0.10 (0.01) | <1x10^-10^ | 0.99 |
|  | PGC+iPSYCH | -0.12 (0.01) | <1x10^-10^ | 1.33 |
| Reading a 9 (NBO) | PGC | -0.02 (0.01) | 0.09 | 0.05 |
|  | iPSYCH | -0.09 (0.01) | <1x10^-10^ | 0.74 |
|  | PGC+iPSYCH | -0.10 (0.01) | <1x10^-10^ | 0.93 |
| Reading s 9 (NARA II) | PGC | -0.04 (0.01) | 7.3x10^-3^ | 0.14 |
|  | iPSYCH | -0.10 (0.01) | <1x10^-10^ | 1.04 |
|  | PGC+iPSYCH | -0.11 (0.01) | <1x10^-10^ | 1.19 |
| Reading a 9 (NARA II) | PGC | -0.04 (0.01) | 6.5x10^-3^ | 0.14 |
|  | iPSYCH | -0.11 (0.01) | <1x10^-10^ | 1.22 |
|  | PGC+iPSYCH | -0.12 (0.01) | <1x10^-10^ | 1.50 |
| Reading s 13 (TOWRE) | PGC | -0.03 (0.02) | 0.03 | 0.11 |
|  | iPSYCH | -0.09 (0.02) | 2.8x10^-9^ | 0.84 |
|  | PGC+iPSYCH | -0.11 (0.02) | <1x10^-10^ | 1.23 |
| NW reading a 9 (NBO) | PGC | -0.03 (0.01) | 0.05 | 0.07 |
|  | iPSYCH | -0.07 (0.01) | 4.2x10^-8^ | 0.53 |
|  | PGC+iPSYCH | -0.09 (0.01) | <1x10^-10^ | 0.77 |
| NW reading s 13 (TOWRE) | PGC | -0.03 (0.02) | 0.05 | 0.09 |
|  | iPSYCH | -0.09 (0.02) | 2.5x10^-8^ | 0.74 |
|  | PGC+iPSYCH | -0.11 (0.02) | <1x10^-10^ | 1.17 |
| Spelling a 7 (NB) | PGC | -0.04 (0.01) | 7.9x10^-3^ | 0.12 |
|  | iPSYCH | -0.09 (0.01) | <1x10^-10^ | 0.86 |
|  | PGC+iPSYCH | -0.11 (0.01) | <1x10^-10^ | 1.17 |
| Spelling a 9 (NB) | PGC | -0.04 (0.01) | 1.4x10^-3^ | 0.18 |
|  | iPSYCH | -0.09 (0.01) | <1x10^-10^ | 0.79 |
|  | PGC+iPSYCH | -0.10 (0.01) | <1x10^-10^ | 1.06 |
| PhonAware 7 (AAT) | PGC | -0.02 (0.01) | 0.22 | 0.03 |
|  | iPSYCH | -0.08 (0.01) | 3.8x10^-9^ | 0.60 |
|  | PGC+iPSYCH | -0.09 (0.01) | <1x10^-10^ | 0.86 |
| Listening c 8 (WOLD) | PGC | -0.02 (0.01) | 0.13 | 0.04 |
|  | iPSYCH | -0.06 (0.01) | 7.9x10^-6^ | 0.37 |
|  | PGC+iPSYCH | -0.08 (0.01) | 1.7x10^-8^ | 0.59 |
| Non-word repetition 8 (CNRep) | PGC | -0.01 (0.01) | 0.70 | 0.003 |
|  | iPSYCH | -0.05 (0.01) | 2.4x10^-4^ | 0.25 |
|  | PGC+iPSYCH | -0.06 (0.01) | 7.6x10^-6^ | 0.37 |
| VIQ 8 (WISC-III) | PGC | -0.03 (0.01) | 0.02 | 0.10 |
|  | iPSYCH | -0.11 (0.01) | <1x10^-10^ | 1.27 |
|  | PGC+iPSYCH | -0.13 (0.01) | <1x10^-10^ | 1.60 |

Abbreviations: LRAs, literacy- and langauge-related abilities; ADHD, Attention-Deficit/Hyperactivity Disorder; R^2^, OLS-regression R^2^; a, accuracy; c, comprehension; s, speed; WORD, Wechsler Objective Reading Dimension; NBO, Nunes, Bryant and Olson (ALSPAC specific instrument); NARA II, The Neale Analysis of Reading Ability- Second Revised British Edition; TOWRE, Test Of Word Reading Efficiency; NW, non-word; NB, Nunes and Bryant (ALSPAC specific instrument); PhonAware, phonemic awareness; AAT, Auditory Analysis Test; WOLD, Wechsler Objective Language Dimensions; CNRep, Children’s Test of Nonword Repetition; VIQ, verbal intelligence quotient; WISC-III, Wechsler Intelligence Scale for Children III; PGC, Psychiatric Genomics Consortium; iPSYCH, The Lundbeck Foundation Initiative for Integrative Psychiatric Research.

ADHD SNPs were selected from GWAS summary statistics based on a *P*-value threshold of 0.1, and alleles were aligned such that the effect allele increased ADHD risk. Children with ADHD were excluded from the ALSPAC sample based on the Development and Wellbeing Assessment(12). LRAs were regressed on ADHD-PGS using ordinary least square regression. Effects were considered as significant if they passed the experiment-wide significance threshold (*P*<0.007).

## Table S7: Genetic correlations of literacy- and language-related measures with educational attainment

| **LRAs** | **r_g_ (SE)** | ***P*** |
| --- | --- | --- |
| Reading a/c 7 (WORD) | 0.65 (0.08) | <1x10^-10^ |
| Reading a 9 (NBO) | 0.57 (0.11) | 2.0x10^-7^ |
| Reading s 9 (NARA II) | 0.77 (0.12) | <1x10^-10^ |
| Reading a 9 (NARA II) | 0.64 (0.08) | <1x10^-10^ |
| Reading s 13 (TOWRE) | 0.80 (0.22) | 3.0x10^-4^ |
| NW reading a 9 (NBO) | 0.61 (0.14) | 2.1x10^-5^ |
| NW reading s 13 (TOWRE) | 0.89 (0.31) | 3.9x10^-3^ |
| Spelling a 7 (NB) | 0.57 (0.08) | <1x10^-10^ |
| Spelling a 9 (NBO) | 0.69 (0.12) | 1.8x10^-8^ |
| PhonAware 7 (AAT) | 0.56 (0.09) | 8.6x10^-10^ |
| Listening c 8 (WOLD) | 0.62 (0.12) | 4.6x10^-7^ |
| Non-word repetition 8 (CNRep) | 0.68 (0.25) | 5.6x10^-3^ |
| VIQ 8 (WISC-III) | 0.82 (0.10) | <1x10^-10^ |

Abbreviations: LRAs, literacy- and language-related abilities; r_g_, genetic correlation; a, accuracy; c, comprehension; s, speed; WORD, Wechsler Objective Reading Dimension; NBO, Nunes, Bryant and Olson (ALSPAC specific instrument); NARA II, The Neale Analysis of Reading Ability- Second Revised British Edition; TOWRE, Test Of Word Reading Efficiency; NW, non-word; NB, Nunes and Bryant (ALSPAC specific instrument); PhonAware, phonemic awareness; AAT, Auditory Analysis Test; WOLD, Wechsler Objective Language Dimensions; CNRep, Children’s Test of Nonword Repetition; VIQ, verbal intelligence quotient; WISC-III, Wechsler Intelligence Scale for Children IIIGenetic correlations were estimated with unconstrained LD-score correlation analyses^34^. Genetic correlations were considered as significant if they passed the experiment-wide significance threshold (*P*<0.007).

## Table S8: Selection of ADHD and EA instruments

| **LRAs** | **ADHD-associated**  **instruments** | | **EA-associated  instruments** | |
| --- | --- | --- | --- | --- |
|  | **Conservative**  **(*P*_thr_<5x10^-8^)** | **Subthreshold**  ***P*_thr_<0.0015)** | **Conservative**  **(*P*_thr_<5x10^-8^)** | **Subthreshold**  ***P*_thr_<0.0015)** |
| Reading a/c 7 (WORD) | 15 | 2,690 | 99 | 4,611 |
| Reading a 9 (NBO) | 15 | 2,690 | 99 | 4,611 |
| Reading s 9 (NARA II) | 15 | 2,689 | 99 | 4,608 |
| Reading a 9 (NARA II) | 15 | 2,689 | 99 | 4,608 |
| Reading s 13 (TOWRE) | 15 | 2,688 | 99 | 4,608 |
| NW reading a 9 (NBO) | 15 | 2,690 | 99 | 4,612 |
| NW reading s 13 (TOWRE) | 15 | 2,688 | 99 | 4,609 |
| Spelling a 7 (NB) | 15 | 2,691 | 99 | 4,613 |
| Spelling a 9 (NB) | 15 | 2,690 | 99 | 4,611 |
| PhonAware 7 (AAT) | 15 | 2,689 | 99 | 4,612 |
| Listening c 8 (WOLD) | 15 | 2,689 | 99 | 4,613 |
| Non-word repetition 8 (CNRep) | 15 | 2,688 | 99 | 4,613 |
| VIQ 8 (WISC-III) | 15 | 2,688 | 99 | 4,612 |

Abbreviations: LRAs, literacy- and language-related abilities; ADHD, Attention-Deficit/Hyperactivity Disorder; EA, educational attainment; *P*_thr_, *P*-value threshold; a, accuracy; c, comprehension; s, speed; WORD, Wechsler Objective Reading Dimension; NBO, Nunes, Bryant and Olson (ALSPAC specific instrument); NARA II, The Neale Analysis of Reading Ability- Second Revised British Edition; TOWRE, Test Of Word Reading Efficiency; NW, non-word; NB, Nunes and Bryant (ALSPAC specific instrument); PhonAware, phonemic awareness; AAT, Auditory Analysis Test; WOLD, Wechsler Objective Language Dimensions; CNRep, Children’s Test of Nonword Repetition; VIQ, verbal intelligence quotient; WISC-III, Wechsler Intelligence Scale for Children III; PGC, Psychiatric Genetics Consortium; iPSYCH, The Lundbeck Foundation Initiative for Integrative Psychiatric Research

ADHD and EA instruments were selected based on ADHD (PGC+iPSYCH) and EA (SSGAC) GWAS summary statistics respectively. Conservative instruments passed the genome-wide significance level (*P*<5x10^-8^), whereas the subthreshold set, containing typically defined instruments, was based on a more lenient *P*-value threshold (*P*<0.0015).

## Table S9: Multivariable regression analysis of polygenic associations between ADHD and literacy- and language-related abilities (standardised)

| **LRAs** | **ADHD (β_ADHD_)**  (ADHD-specific effects independent of EA) | | | | | | **EA (β_EA_)**  (ADHD genetic effects shared with EA)^1^ | | | | | |
| --- | --- | --- | --- | --- | --- | --- | --- | --- | --- | --- | --- | --- |
|  | **Conservative instruments (*P*_thr_<5x10^-8^)** | | | **Subthreshold instruments (*P*_thr_<0.0015)** | | | **Conservative instruments (*P*_thr_<5x10^-8^)** | | | **Subthreshold instruments (*P*_thr_<0.0015)** | | |
|  | **β (SE)** | ***P*** | ***P_het_*** | **β (SE)** | ***P*** | ***P_het_*** | **β (SE)** | ***P*** | ***P_het_*** | **β (SE)** | ***P*** | ***P_het_*** |
| Reading a/c 7 (WORD) | -0.21 (0.23) | 0.39 | - | -0.06 (0.01) | 8.3x10^-6^ | - | 0.12 (0.27) | 0.66 | - | -0.15 (0.02) | 1.5x10^-9^ | - |
| Reading a 9 (NBO) | -0.46 (0.16) | 0.01 | - | -0.05 (0.01) | 1.4x10^-4^ | - | -0.27 (0.19) | 0.18 | - | -0.11 (0.02) | 7.0x10^-6^ | - |
| Reading s 9 (NARA II) | -0.44 (0.23) | 0.07 | - | -0.06 (0.01) | 3.4x10^-5^ | - | -0.30 (0.27) | 0.29 | - | -0.12 (0.03 | 2.2x10^-6^ | - |
| Reading a 9 (NARA II) | -0.51 (0.22) | 0.04 | - | -0.05 (0.01) | 3.8x10^-4^ | - | -0.27 (0.26) | 0.33 | - | -0.15 (0.03) | 3.4x10^-9^ | - |
| Reading s 13 (TOWRE) | -0.63 (0.25) | 0.02 | - | -0.07 (0.02) | 2.1x10^-5^ | - | -0.48 (0.29) | 0.12 | - | -0.11 (0.03) | 2.3x10^-4^ | - |
| NW reading a 9 (NBO) | -0.56 (0.18) | 0.01 | - | -0.04 (0.01) | 8.5x10^-4^ | - | -0.26 (0.21) | 0.25 | - | -0.11 (0.02) | 4.6x10^-6^ | - |
| NW reading s 13 (TOWRE) | -0.32 (0.30) | 0.31 | - | -0.06 (0.02) | 1.4x10^-4^ | - | -0.28 (0.35) | 0.45 | - | -0.10 (0.03) | 2.4x10^-4^ | - |
| Spelling a 7 (NB) | 0.004 (0.21) | 0.98 | - | -0.08 (0.01) | 9.7x10^-9^ | - | 0.34 (0.25) | 0.21 | - | -0.09 (0.02) | 1.5x10^-4^ | - |
| Spelling a 9 (NB) | -0.34 (0.14) | 0.03 | - | -0.07 (0.01) | 6.4x10^-7^ | - | -0.27 (0.17) | 0.14 | - | -0.11 (0.02) | 1.3x10^-5^ | - |
| PhonAware 7 (AAT) | -0.25 (0.26) | 0.34 | - | -0.04 (0.01) | 0.002 | - | 0.20 (0.31) | 0.53 | - | -0.16 (0.02) | <1x10^-10^ | - |
| Listening c 8 (WOLD) | -0.05 (0.13) | 0.74 | - | -0.03 (0.01) | 0.02 | - | 0.33 (0.16) | 0.05 | - | -0.13 (0.03) | 3.9x10^-7^ | - |
| Non-word repetition 8 (CNRep) | -0.20 (0.25) | 0.42 | - | -0.03 (0.01) | 0.02 | - | 0.08 (0.29) | 0.79 | - | -0.11 (0.03) | 1.2x10^-5^ | - |
| VIQ 8 (WISC-III) | -0.30 (0.27) | 0.29 | - | -0.06 (0.01) | 5.2x10^-5^ | - | 0.18 (0.32) | 0.57 | - | -0.17 (0.02) | <1x10^-10^ | - |
| Pooled reading | -0.55 (0.16) | 7.2x10^-4^ | 0.13 | -0.06 (0.01) | 1.3x10^-6^ | 0.69 | 0.34 (0.19) | 0.08 | 0.19 | -0.12 (0.02) | 5.0x10^-8^ | 0.09 |
| Pooled spelling | -0.22 (0.20) | 0.27 | 0.01 | -0.07 (0.01) | 8.4x10^-9^ | 0.31 | 0.01 (0.31) | 0.97 | 3.0x10^-4^ | -0.09 (0.02) | 1.3x10^-5^ | 0.40 |
| Pooled LRAs | -0.27 (0.13) | 0.03 | 0.01 | -0.05 (0.01) | 1.4x10^-6^ | 0.05 | 0.02 (0.16) | 0.91 | 0.002 | -0.12 (0.02) | <1x10^-10^ | 0.002 |

1. ADHD genetic effects shared with EA as assessed through EA genetic effects of ADHD-associated variants

Abbreviations: LRAs, literacy- and language-related abilities; ADHD, Attention-Deficit/Hyperactivity Disorder; EA, educational attainment; *P*_thr_, *P*-value threshold; *P*_het_, Heterogeneity *P*-value; a, accuracy; c, comprehension; s, speed; WORD, Wechsler Objective Reading Dimension; NBO, Nunes, Bryant and Olson (ALSPAC specific instrument); NARA II, The Neale Analysis of Reading Ability- Second Revised British Edition; TOWRE, Test Of Word Reading Efficiency; NW, non-word; NB, Nunes and Bryant (ALSPAC specific instrument); PhonAware; phonemic awareness; AAT, Auditory Analysis Test; WOLD, Wechsler Objective Language Dimensions; CNRep, Children’s Test of Nonword Repetition; VIQ, verbal intelligence quotient; WISC-III, Wechsler Intelligence Scale for Children III; MVR, Multivariable regression

Sets of conservative (P<5x10^-8^) and subthreshold (P<0.0015) ADHD instruments were extracted from ADHD (PGC+iPSYCH), EA (SSGAC) and LRAs (ALSPAC) GWAS summary statistics. ADHD-specific effects independent of EA (β_ADHD_) and ADHD effects shared with EA (β_EA_) on LRAs were estimated with MVRs. To compare the magnitude of MVR estimates, analyses were conducted using standardised regression estimates (Supplementary Methods). β_ADHD_ estimates measure the change in LRA Z-score per Z-score in ADHD liability. β_EA_ estimates measure the change in LRA Z-scores per Z-score in missing school years. MVR estimates based on raw genetic effect estimates are provided in Table 3. Pooled estimates for reading, spelling and global LRA measures (Table 1) were obtained through random-effects meta-regression. Effects were considered significant if they passed the experiment-wide significance threshold (*P*<0.007).

## Table S10: Comparison of ADHD-specific MVR effects on literacy-related abilities versus other LRAs

| **ADHD instruments** | **Effect** | **Beta (SE)** | ***P*** | ***P*_res_ _het_** |
| --- | --- | --- | --- | --- |
| Conservative (*P*_thr_<5x10^-8^) | Reading | -0.300 (0.082) | 2x10^-4^ | 0.054 |
|  | Other LRAs (Moderator) | 0.150 (0.059) | 0.011 |  |
| Subthreshold  (*P*_thr_<0.0015) | Reading | -0.024 (0.006) | 1x10^-4^ | 0.054 |
|  | Other LRAs (Moderator) | -0.003 (0.004) | 0.427 |  |
|  | Spelling | -0.036 (0.006) | 2x10^-9^ | 0.56 |
|  | Other LRAs (Moderator) | 0.012 (0.004) | 0.001 |  |
|  | Reading+spelling | -0.035 (0.006) | 5x10^-8^ | 0.22 |
|  | Other LRAs (Moderator) | 0.012 (0.005) | 0.016 |  |

Abbreviations: ADHD, Attention-Deficit/Hyperactivity Disorder; LRAs, language- and literacy-related abilities; *P_res_* _het_ -Evidence for residual effect heterogeneity; *P*_thr_, *P*-value threshold

ADHD-specific effect differences on LRAs were compared using contrasts within a random-effects meta-regression model, based on all LRAs studied, while accounting for phenotypic inter-correlations. Moderator effects were considered significant at a significance level of 0.05.

## Table S11: Multivariable regression analysis of polygenic associations between EA and literacy- and language-related abilities (standardised)

| **LRAs** | **EA (β_EA_)**  (EA-specific effects independent of ADHD) | | | | | | **ADHD (β_ADHD_)**  (EA genetic effects shared with ADHD )^1^ | | | | | | | | |
| --- | --- | --- | --- | --- | --- | --- | --- | --- | --- | --- | --- | --- | --- | --- | --- |
|  | **Conservative instruments (*P*_thr_<5x10^-8^)** | | | **Subthreshold instruments (*P*_thr_<0.0015)** | | | **Conservative instruments (*P*_thr_<5x10^-8^)** | | | | **Subthreshold instruments (*P*_thr_<0.0015)** | | | |  |
|  | **β (SE)** | ***P*** | ***P_het_*** | **β (SE)** | ***P*** | ***P_het_*** | | **β (SE)** | ***P*** | ***P_het_*** | | **β (SE)** | ***P*** | ***P_het_*** |  |
| Reading a/c 7 (WORD) | -0.16 (0.03) | 4.4x10^-6^ | - | -0.11 (0.01) | <1x10^-10^ | - | | -0.27 (0.14) | 0.07 | - | | -0.16 (0.03) | 6.9x10^-9^ | - |  |
| Reading a 9 (NBO) | -0.14 (0.03) | 5.8x10^-5^ | - | -0.09 (0.01) | <1x10^-10^ | - | | -0.24 (0.14) | 0.09 | - | | -0.16 (0.03) | 1.4x10^-8^ | - |  |
| Reading s 9 (NARA II) | -0.21 (0.04) | 1.3x10^-7^ | - | -0.11 (0.01) | <1x10^-10^ | - | | -0.13(0.16) | 0.41 | - | | -0.15 (0.03) | 2.8x10^-7^ | - |  |
| Reading a 9 (NARA II) | -0.18 (0.04) | 4.0x10^-6^ | - | -0.11 (0.01) | <1x10^-10^ | - | | -0.42 (0.16) | 0.01 | - | | -0.17 (0.03) | 8.9x10^-8^ | - |  |
| Reading s 13 (TOWRE) | -0.17 (0.04) | 3.7x10^-5^ | - | -0.09 (0.01) | <1x10^-10^ | - | | -0.09 (0.17) | 0.59 | - | | -0.14 (0.03) | 6.0x10^-5^ | - |  |
| NW reading a 9 (NBO) | -0.13 (0.03) | 1.3x10^-4^ | - | -0.09 (0.01) | <1x10^-10^ | - | | -0.38 (0.14) | 0.01 | - | | -0.09 (0.03) | 0.001 | - |  |
| NW reading s 13 (TOWRE) | -0.13 (0.03) | 1.3x10^-4^ | - | -0.09 (0.01) | <1x10^-10^ | - | | -0.20 (0.14) | 0.16 | - | | -0.15 (0.03) | 7.9x10^-6^ | - |  |
| Spelling a 7 (NB) | -0.17 (0.04) | 1.5x10^-5^ | - | -0.09 (0.01) | <1x10^-10^ | - | | -0.26 (0.16) | 0.10 | - | | -0.16 (0.03) | 1.4x10^-8^ | - |  |
| Spelling a 9 (NB) | -0.13 (0.04) | 3.8x10^-4^ | - | -0.10 (0.01) | <1x10^-10^ | - | | -0.29(0.15) | 0.06 | - | | -0.13 (0.03) | 4.8x10^-6^ | - |  |
| PhonAware 7 (AAT) | -0.13 (0.03) | 2.7x10^-4^ | - | -0.09 (0.01) | <1x10^-10^ | - | | -0.25 (0.14) | 0.09 | - | | -0.13 (0.03) | 3.9x10^-6^ | - |  |
| Listening c 8 (WOLD) | -0.19 (0.03) | 6.6x10^-8^ | - | -0.08 (0.01) | <1x10^-10^ | - | | 0.04 (0.14) | 0.76 | - | | -0.08 (0.03) | 0.005 | - |  |
| Non-word repetition 8 (CNRep) | -0.13 (0.04) | 8.4x10^-4^ | - | -0.08 (0.01) | <1x10^-10^ | - | | 0.05 (0.16) | 0.73 | - | | -0.04 (0.03) | 0.15 | - |  |
| VIQ 8 (WISC-III) | -0.24 (0.03) | 1.9x10^-10^ | - | -0.13 (0.01) | <1x10^-10^ | - | | -0.06 (0.15) | 0.71 | - | | -0.18 (0.03) | 5.7x10^-9^ | - |  |
| Pooled reading | -0.14 (0.03) | 5.0x10^-7^ | 0.08 | -0.10 (0.01) | <1x10^-10^ | <0.001 | | -0.23 (0.12) | 0.06 | 0.12 | | -0.14 (0.03) | 1.2x10^-7^ | 0.07 |  |
| Pooled spelling | -0.15 (0.04) | 2.9x10^-5^ | 0.16 | -0.10 (0.01) | <1x10^-10^ | 0.34 | | -0.28 (0.14) | 0.06 | 0.81 | | -0.15 (0.03) | 1.6x10^-7^ | 0.15 |  |
| Pooled LRAs | -0.15 (0.02) | 1.2x10^-10^ | 0.02 | -0.09 (0.01) | <1x10^-10^ | <0.001 | | -0.06 (0.09) | 0.50 | 0.18 | | -0.11 (0.02) | 4.0x10^-7^ | <0.001 |  |

1.EA genetic effects shared with ADHD as assessed through ADHD genetic effect estimates of EA-associated variants

Abbreviations: LRAs, literacy- and language-related abilities; ADHD, Attention-Deficit/Hyperactivity Disorder; EA, educational attainment; *P*_thr_, *P*-value threshold; *P*_het_, Heterogeneity *P*-value; a, accuracy; c, comprehension; s, speed; WORD, Wechsler Objective Reading Dimension; NBO, Nunes, Bryant and Olson (ALSPAC specific instrument); NARA II, The Neale Analysis of Reading Ability- Second Revised British Edition; TOWRE, Test Of Word Reading Efficiency; NW, non-word; NB, Nunes and Bryant (ALSPAC specific instrument); PhonAware, phonemic awareness; AAT, Auditory Analysis Test; WOLD, Wechsler Objective Language Dimensions; CNRep, Children’s Test of Nonword Repetition; VIQ, verbal intelligence quotient; WISC-III, Wechsler Intelligence Scale for Children III;; MVR - Multivariable regression.

Sets of conservative (P<5x10^-8^) and subthreshold (P<0.0015) EA instruments were extracted from EA (SSGAC), ADHD (PGC+iPSYCH), and LRAs (ALSPAC) GWAS summary statistics. EA-specific effects independent of ADHD (β_EA_) and EA effects shared with ADHD (β_ADHD_) on LRAs were estimated with MVRs. To compare the magnitude of β_ADHD_ and β_EA_, MVR analyses were conducted using standardised regression estimates (Supplementary Methods). β_EA_ estimates measure the change in LRA Z-scores per Z-score in missing school years. β_ADHD_ estimates measure the change in LRA Z-score per Z-score in ADHD liability. Pooled estimates for reading, spelling and global LRA measures (Table 1) were obtained through random-effects meta-regression. Effects were considered significant if they passed the experiment-wide significance threshold (*P*<0.007).

##

## Table S12: Association between polygenic ADHD risk and sample-dropout

| **Outcome** | **ADHD sample** | **OR (SE)** | ***P*** | **McFadden's pseudo R^2^** |
| --- | --- | --- | --- | --- |
| Sample drop-out for reading accuracy and comprehension at age 7 (WORD) | PGC | -0.995 (0.005) | 0.36 | 8.6x10^-5^ |
|  | iPSYCH | 1.029 (0.005) | 5.0x10^-9^ | 3.5x10^-3^ |
|  | PGC+iPSYCH | 1.028 (0.005) | 1.4x10^-8^ | 3.3x10^-3^ |

Abbreviations: ADHD, Attention-Deficit/Hyperactivity Disorder; WORD, Wechsler Objective Reading Dimension; PGC, Psychiatric Genomics Consortium; iPSYCH, The Lundbeck Foundation Initiative for Integrative Psychiatric Research

A yes/no variable indicating sample drop-out for reading accuracy and comprehension at age 7 (WORD) was generated. ADHD SNPs were selected based on a *P*-value threshold of 0.1, and alleles were aligned such that the effect allele increased ADHD risk. Sample drop-out was regressed on polygenic ADHD risk scores using logistic regression. The experiment-wide significance threshold is *P*<0.007.

## Table S13: Multivariable regression analysis of polygenic associations between ADHD, sample drop-out and literacy- and language-related abilities

|  | **ADHD (β_ADHD_)**  (ADHD-specific effects independent of EA) | | | **EA (β_EA_)**  (EA genetic effects of ADHD-associated variants)^1^ | | |
| --- | --- | --- | --- | --- | --- | --- |
| **ADHD instruments** | **OR (SE)** | **β (SE)** | ***P*** | **OR (SE)** | **β (SE)** | ***P*** | |
| Conservative  (*P*_thr_<5x10^-8^) | 0.99 (0.29) | -0.01 (0.30) | 0.96 | 50.0 (133.7) | 3.91 (2.68) | 0.17 | |
| Subthreshold  (*P*_thr_<0.0015) | 1.05 (0.02) | 0.05 (0.01) | 3.7x10^-4^ | 2.83 (0.54) | 1.04 (0.19) | 7.3x10^-8^ | |

1. ADHD genetic effects shared with EA as assessed through EA genetic effect estimates of ADHD-associated variants

Abbreviations: ADHD, Attention-Deficit/Hyperactivity Disorder; EA, educational attainment; *P*_thr_, *P*-value threshold; MVR - Multivariable regression

Sets of conservative (*P*<5x10^-8^) and subthreshold (*P*<0.0015) ADHD instruments were extracted from ADHD (PGC+iPSYCH), EA (SSGAC) and sample drop-out for reading accuracy and comprehension at age 7 (WORD) (ALSPAC) GWAS summary statistics. ADHD-specific effects independent of EA (β_ADHD_) and ADHD effects shared with EA (β_EA_) on LRAs were estimated with MVRs. β_ADHD_ estimates measure the change in liability to drop-out per log odds increase in ADHD liability. β_EA_ estimates measure the change in liability to drop-out per missing school year. The experiment-wide significance threshold is *P*<0.007.

# Supplementary Figures

##
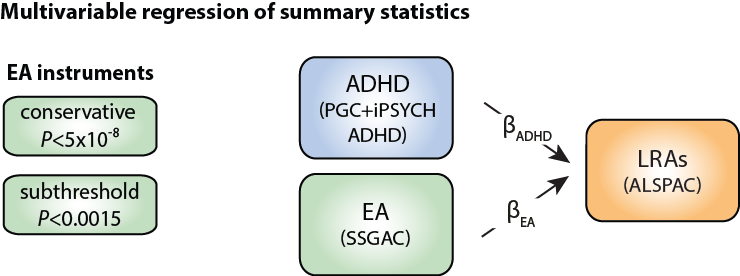


## Figure S1: Multivariable regression analysis of polygenic associations between EA and literacy- and language-related abilities

Abbreviations: ADHD, Attention-Deficit/Hyperactivity Disorder; EA, educational attainment; LRAs, literacy- and language-related abilities; MVR - Multivariable regression

Sets of conservative (*P*<5x10^-8^) and subthreshold (*P*<0.0015) EA instruments were extracted from ADHD (PGC+iPSYCH), EA (SSGAC) and LRAs (ALSPAC) GWAS summary statistics. EA-specific effects independent of ADHD (β_EA_) and EA effects shared with ADHD (β_ADHD_) on LRAs were estimated with MVRs. EA effects shared with ADHD were assessed through ADHD genetic effect estimates of EA-associated variants.
